# Scaffolding Protein ENH Promotes Tumor Angiogenesis and Growth Through Macrophage Recruitment and Polarization

**DOI:** 10.1002/advs.202416476

**Published:** 2025-06-19

**Authors:** Yueli Shi, Zhiyong Xu, Huan Wang, Bufu Tang, Nueraili Maihemuti, Xinyuan Jiang, Xiuying Chen, Mingshu Xiao, Sujing Jiang, Yun Xu, Peng Xiao, Jiangnan Zhao, Kaiyue Zhang, Mengshu Li, Kai Wang

**Affiliations:** ^1^ Department of Respiratory and Critical Care Medicine Center for Oncology Medicine the Fourth Affiliated Hospital of School of Medicine and International School of Medicine International Institutes of Medicine Zhejiang University Yiwu 322000 China; ^2^ Zhejiang Key Laboratory of Precision Diagnosis and Treatment for Lung Cancer Yiwu 322000 China; ^3^ Department of Respiratory & Critical Care Medicine The First Affiliated Hospital of Nanjing Medical University Nanjing 210000 China; ^4^ Department of Interventional Radiology Zhongshan Hospital Fudan University Shanghai 200032 China; ^5^ Department of Obstetrics and Gynecology Center for Reproductive Medicine the Fourth Affiliated Hospital of School of Medicine and International School of Medicine International Institutes of Medicine Zhejiang University Yiwu 322000 China; ^6^ Department of Gastroenterology The Second Affiliated Hospital of Wenzhou Medical University Wenzhou 325000 China; ^7^ Sir Run Run Shaw Hospital Zhejiang University School of Medicine Hangzhou 310016 China

**Keywords:** angiogenesis, ENH protein, lung adenocarcinoma, tumor‐associated macrophages, YAP signaling

## Abstract

Angiogenesis is vital for tumor growth and metastasis, with tumor‐associated macrophages (TAMs) being key pro‐angiogenic cells recruited by tumor‐secreted chemokines. High levels of TAMs contribute to tumor progression and antiangiogenic therapy resistance. Therefore, intensive study of the regulatory mechanisms of TAMs recruitment during tumor development is important for the discovery of new antitumor and antiangiogenic therapeutic strategies. Here, we found that in lung adenocarcinoma (LUAD), ENH levels positively correlated with microvessel density and TAMs infiltration. Further exploration revealed that ENH promoted LUAD angiogenesis and growth by stimulating TAMs recruitment and M2 polarization. Mechanistically, ENH in LUAD induced YAP nuclear aggregation to promote CCL5 transcription, thereby increasing monocyte chemotaxis and ultimately increasing TAMs infiltration and M2 polarization. Besides, we found that ENH interacted with YAP through LIM domains, which significantly triggered the formation of YAP‐KPNA2 complexes. Consequently, YAP is imported into the nucleus by KPNA2 and then promoted CCL5 transcription. Notably, ENH knockdown also significantly increased the chemosensitivity. Together, ENH functions in LUAD cells to mediate macrophage infiltration and M2 polarization, which in turn promotes tumor angiogenesis and growth, and targeting ENH offers a promising target for antiangiogenic therapy through immune modulation.

## Graphical abstract

In tumor cells, ENH prompts the interaction between YAP and KPNA2. This interaction enables YAP to translocate into the nucleus, where it enhances the transcription of CCL5. Consequently, this up‐regulation of CCL5 drives the infiltration of tumor‐associated macrophages (TAMs) and their polarization toward the M2 phenotype, ultimately facilitating tumor angiogenesis and growth.

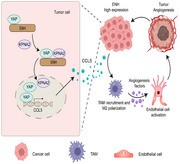

## 1 Introduction

Lung cancer is the leading cause of cancer‐related mortality worldwide, with ≈1.8 million deaths annually. Non‐small cell lung cancer (NSCLC) accounts for over 85% of all lung cancer types, with lung adenocarcinoma (LUAD) being the most common histological subtype.^[^ ^1^ ^]^ In recent years, advancements in diagnosis and treatment strategies have improved the clinical outcomes of patients with LUAD. However, the mortality rate remains high, with a 5‐year survival rate of <20%.^[^ ^2^, ^3^ ^]^ Angiogenesis is essential for the growth and metastasis of LUAD and other malignant tumors. During the sustained growth of tumors, neovascularization provides sufficient oxygen and nutrients for tumors and removes metabolic waste and carbon dioxide. Without this vascular support, most solid tumors will not grow beyond 2 mm.^[^ ^3^, ^4^, ^5^ ^]^ Tumor angiogenesis is a complex process involving multiple cellular and molecular interactions. Typically, endothelial cells start to proliferate and migrate under the stimulation of pro‐angiogenic factors and finally form new blood vessels. During this process, tumor cells and stromal cells such as macrophages are responsible for secreting pro‐angiogenic factors.^[^ ^6^ ^]^ Up to now, a large number of pro‐angiogenic signals have been identified, among which the VEGF/ VEGFR signaling pathway is the most crucial one.^[^ ^7^, ^8^ ^]^ Studies have shown that increased vessel density and elevated levels of VEGF, correlate with LUAD progression.^[^ ^9^, ^10^ ^]^ Consequently, inhibiting angiogenesis can effectively inhibit LUAD development. Tumor‐associated macrophages (TAMs) are the most abundant immune cell population in the tumor microenvironment (TME) and play critical roles in immunosuppression, pro‐angiogenesis, and tumor promotion.^[^ ^11^ ^]^ Clinical studies indicate that high levels of TAMs are associated with resistance to antiangiogenic therapies and poor survival outcomes in cancers.^[^ ^12^, ^13^ ^]^ TAMs are derived from monocytes, which are recruited from the bone marrow into the tumor by chemoattractants in TME, such as CSF‐1, CCL2, CCL3, CCL4, and CCL5.^[^ ^14^, ^15^ ^]^ Pre‐clinical studies have demonstrated that inhibiting TAM infiltration into tumors significantly attenuates tumor angiogenesis, growth, and metastasis.^[^ ^16^, ^17^, ^18^ ^]^ Given this, clinical trials targeting TAMs are being launched one after another. For example, a series of drugs targeting CSF‐1/CSF‐R1 and CCL2/CCR2 signaling pathways have been developed to inhibit TAMs infiltration for anti‐tumor treatment. However, currently, these drugs haven't been applied clinically due to poor efficacy or severe toxicity.^[^ ^19^ ^]^ Thus, it's significant to deeply explore the mechanism of regulating TAMs infiltration to develop more targeted and effective new treatment strategies.

Macrophages are known to differentiate into classically activated M1‐type and alternatively activated M2‐type. In tumors, TAMs usually present an M2 state, facilitating tumor angiogenesis and malignancy.^[^ ^20^, ^21^ ^]^ Thus, suppressing M2 activation, depleting M2 TAMs, or inducing a pro‐inflammatory M1 state can effectively diminish TAM‐mediated tumor progression.^[^ ^22^, ^23^ ^]^ Although TAMs have recently emerged as promising targets for cancer treatment, the mechanisms underlying their role in angiogenesis and malignant tumor progression are not fully understood.

As key anchor molecules of intracellular signal transduction, scaffolding proteins play an important role in tumorigenesis and malignant progression.^[^ ^24^ ^]^ Recent studies have also implicated these proteins in the regulation of TME. The scaffolding protein ENH (PDLIM5) is an important member of the PDZ‐LIM family, which structurally contains an N‐terminal PDZ domain and three C‐terminal LIM domains.^[^ ^25^ ^]^ Through the PDZ and LIM domains, ENH binds to a variety of different proteins to carry out diverse functions, including tumorigenesis.^[^ ^26^, ^27^ ^]^ For instance, as reported in the study,^[^ ^28^ ^]^ ENH interacts with CREB by means of the LIM domain. This interaction triggers the phosphorylation of CREB, and subsequently, ENH gets involved in the regulation of cardiomyocyte function. Additionally, ENH can also bind to ID2 via the LIM domain, thereby exerting an inhibitory effect on cell proliferation.^[^ ^29^ ^]^ ENH is upregulated in various cancers, and its high levels are associated with poor prognosis. Genetic suppression of ENH efficiently attenuates the migration, invasion, and proliferation of several cancer cells.^[^ ^30^ ^]^ Additionally, ENH upregulation has been linked to tumor resistance to EGFR‐TKIs, as seen in NSCLC.^[^ ^31^ ^]^ These results indicate that targeting ENH could be an effective anti‐tumor strategy. Researchers are currently experimenting with nanomaterials to deliver ENH siRNA for antitumor treatment.^[^ ^32^, ^33^ ^]^

Our previous study reported that ENH is upregulated in NSCLC and promotes tumor metastasis by inhibiting STUB1‐mediated degradation of SMAD3.^[^ ^34^ ^]^ However, it is unclear whether ENH plays a role in regulating TME in lung cancer progression. In this study, we revealed for the first time that ENH promoted YAP nuclear translocation by enhancing the interaction of YAP with KPNA2, leading to increased transcription of the chemokine CCL5. This process facilitates TAMs recruitment and M2 polarization, ultimately promoting tumor angiogenesis and growth.

## 2 Result

### 2.1 ENH is Closely Linked to MVD Levels in LUAD Tumor Tissue and Plays a Significant Role in Promoting Angiogenesis

**Figure 1:**
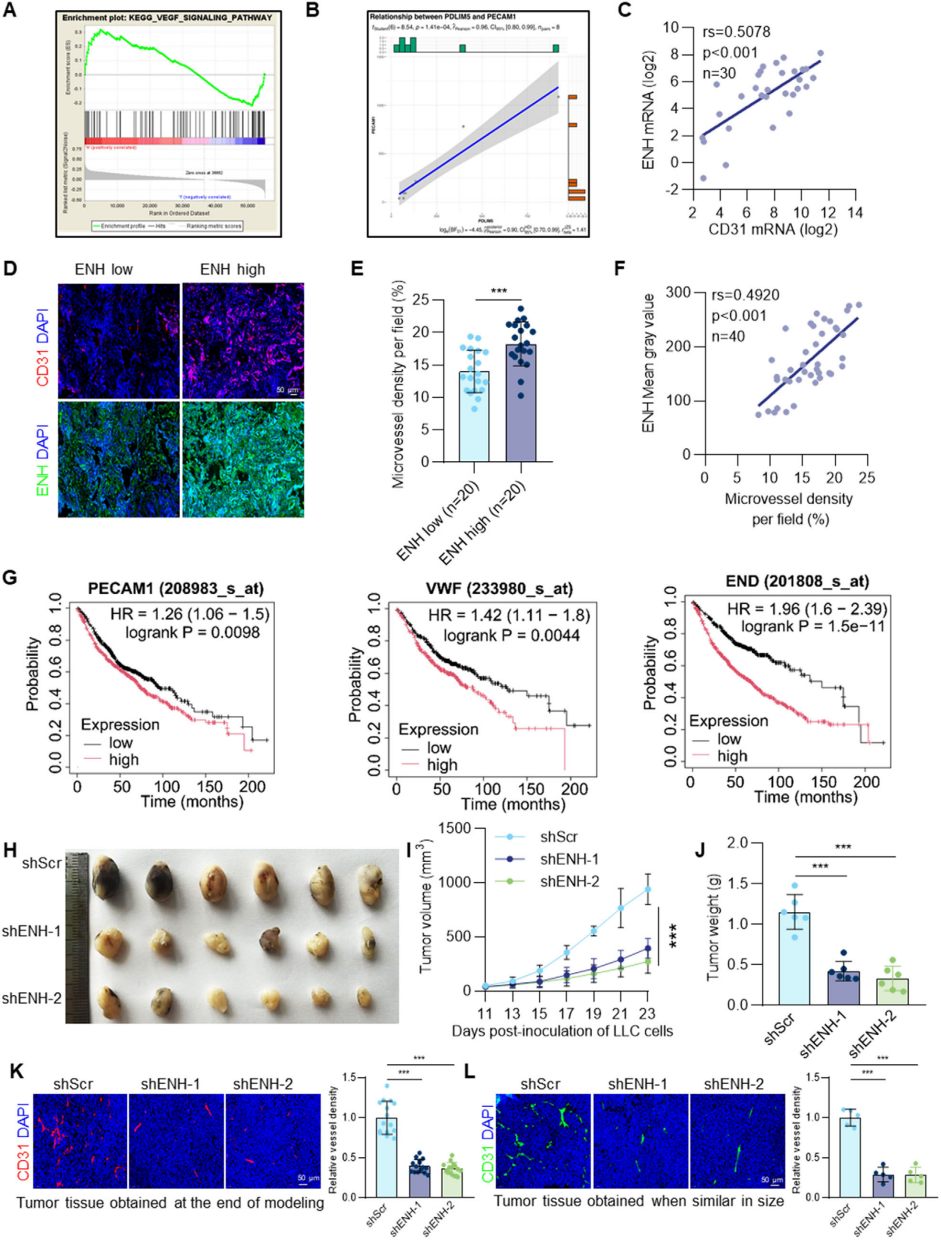
ENH is strongly associated with MVD levels in LUAD tumor tissue and promotes angiogenesis. A) GSEA revealed that ENH expression mainly affected the VEGF signaling pathway. B) The correlation between ENH and angiogenesis marker PECAM1 in LUAD was analyzed using the GEO datasets (GSE85841). C) Identification of the correlation between ENH and CD31 mRNAs in 30 LUAD tissues by qPCR. D) Representative images of IF staining for ENH and CD31 in human LUAD tissues were shown. E) The microvessel counts were quantified based on the IF results, and the results were presented in two groups according to low and high ENH expression (*n* = 20). F) Identification of the correlation between ENH and MVD based on the IF results (*n* = 40). G) Kaplan–Meier analysis of OS of LUAD patients was conducted based on endothelial‐specific marker expression levels (CD31, vWF, END). H) Tumor tissues from each group collected 23 days after injection of LLC cells (*n* = 6). I) Subcutaneous tumor growth of LLC cells stably transduced with control shRNA (shScr) or shRNA targeting ENH (shENH‐1, shENH‐2) was observed (*n* = 6). J) Tumor weight of LLC murine model in ENH knockdown or control group at day 23 after tumor injection (*n* = 6). K) Representative images of IF staining for CD31 in subcutaneous tumor tissues obtained from ENH knockdown and control groups at the end of modeling. Quantification of CD31+ vessel density was shown (*n* = 15). L) Representative images of IF staining for CD31 of subcutaneous tumor tissues of ENH knockdown and control groups were obtained when tumors were similar in size. Quantification of CD31+ vessel density was shown (*n* = 5).

To investigate the role of ENH in regulating the LUAD TME, we conducted gene set enrichment analysis (GSEA) using RNA sequencing data of LUAD from TCGA database. The results showed that ENH positively correlated with the VEGF signaling pathway (**Figure** **1**A). As the VEGF pathway is the main signal promoting tumor angiogenesis, we then analyzed the expression correlation between ENH and vascular endothelial‐specific marker PECAM1 (CD31). We found a positive correlation between ENH and PECAM1 expression levels in LUAD and other malignant tumors (Figure 1B; Figure S1, Supporting Information). This correlation was confirmed by analyzing the mRNA of ENH and CD31 in the LUAD tissues from our study (Figure 1C). Using CD31 antibodies to label tumor vasculature and analyzing MVD, we found that MVD was higher in LUAD tissues with high ENH expression than in those with low ENH expression (Figure 1D,E). A significant correlation was also observed between ENH expression and MVD levels (Figure 1F). Furthermore, survival analysis showed that LUAD patients with high MVD had a shorter overall survival (OS) time compared to those with low MVD, as evidenced by analysis of the relationship between the expression of a series of endothelial‐specific markers CD31, vWF, END, and OS (Figure 1G). These results suggest that ENH promotes tumor angiogenesis in LUAD. We then constructed cells with stable ENH overexpression or knockdown to assess the effect of ENH on tumor development. While ENH knockdown did not alter the in vitro growth or apoptosis rate of LUAD cells (Figure S2A–I, Supporting Information), it inhibited tumor growth in mouse xenografts (Figure 1H–J). Conversely, ENH overexpression promoted xenograft tumor growth (Figure S3A–C, Supporting Information). Given the positive correlation between ENH and MVD, we examined ENH's role in angiogenesis. The results showed that ENH overexpression significantly increased MVD in xenograft tumor tissues, while ENH knockdown decreased it (Figure S3D,E, Supporting Information; Figure 1K). To determine whether the reduced blood vessel density is due to reduced tumor volume or whether reduced tumor volume is due to reduced blood vessel density. We obtained similarly sized tumors during xenografting to assess MVD levels and found a consistent result of significant downregulation of MVD levels in the ENH knockdown groups (Figure 1L). The above results suggest that tumor cell ENH promotes tumor growth by regulating angiogenesis.

### 2.2 Tumor‐Infiltrating TAMs are Indispensable for ENH‐Driven Tumor Growth and Angiogenesis

To investigate the potential mechanisms of ENH in regulating angiogenesis, we first examined the expression of major pro‐angiogenic factors in ENH‐knockdown tumor cells. Interestingly, ENH knockdown did not noticeably alter their expression (Figure S3F, Supporting Information). We also explored whether altering ENH expression affected the pro‐angiogenic function of tumor cells. Surprisingly, neither the supernatants of ENH‐overexpressing nor knockdown LUAD cells affected the migration, proliferation, and tube formation capacity of endothelial cells when compared to the control group (Figure S4A–F, Supporting Information). Therefore, we hypothesized that ENH promotes tumor angiogenesis through an indirect mechanism.

TAMs are known to play a crucial role in tumor angiogenesis and progression. Since TAMs are derived from infiltrating monocytes, we hypothesized that ENH affected the angiogenesis and progression of LUAD by regulating the recruitment of monocyte‐derived TAMs. Analysis revealed a significant positive correlation between ENH expression and TAMs/monocytes content in the TEM of LUAD (Figure S5A,B, Supporting Information). Further, ENH expression correlated with the expression of macrophage/monocyte markers in TCGA database (Figure S5C, Supporting Information). Additionally, staining of human LUAD tissue with anti‐CD68 (macrophage marker) antibodies confirmed higher TAMs infiltration in LUAD tissues with high ENH expression compared to those with low ENH expression (Figure S5D,E, Supporting Information). This experiment also confirmed a positive correlation between ENH expression and TAMs infiltration (Figure S5F, Supporting Information). Apart from these, CD68 and CD14 expression were also significantly positively correlated with the expression of PECAM1 (Figure S5G, Supporting Information), and patients with high CD68 expression had a significantly lower OS rate compared to patients with low CD68 expression (Figure S5H, Supporting Information). By analyzing the scRNA seq‐data of GES131907, we also found that ENH has a potent positive impact on the infiltration of macrophages (Figure S6A–D, Supporting Information).

**Figure 2:**
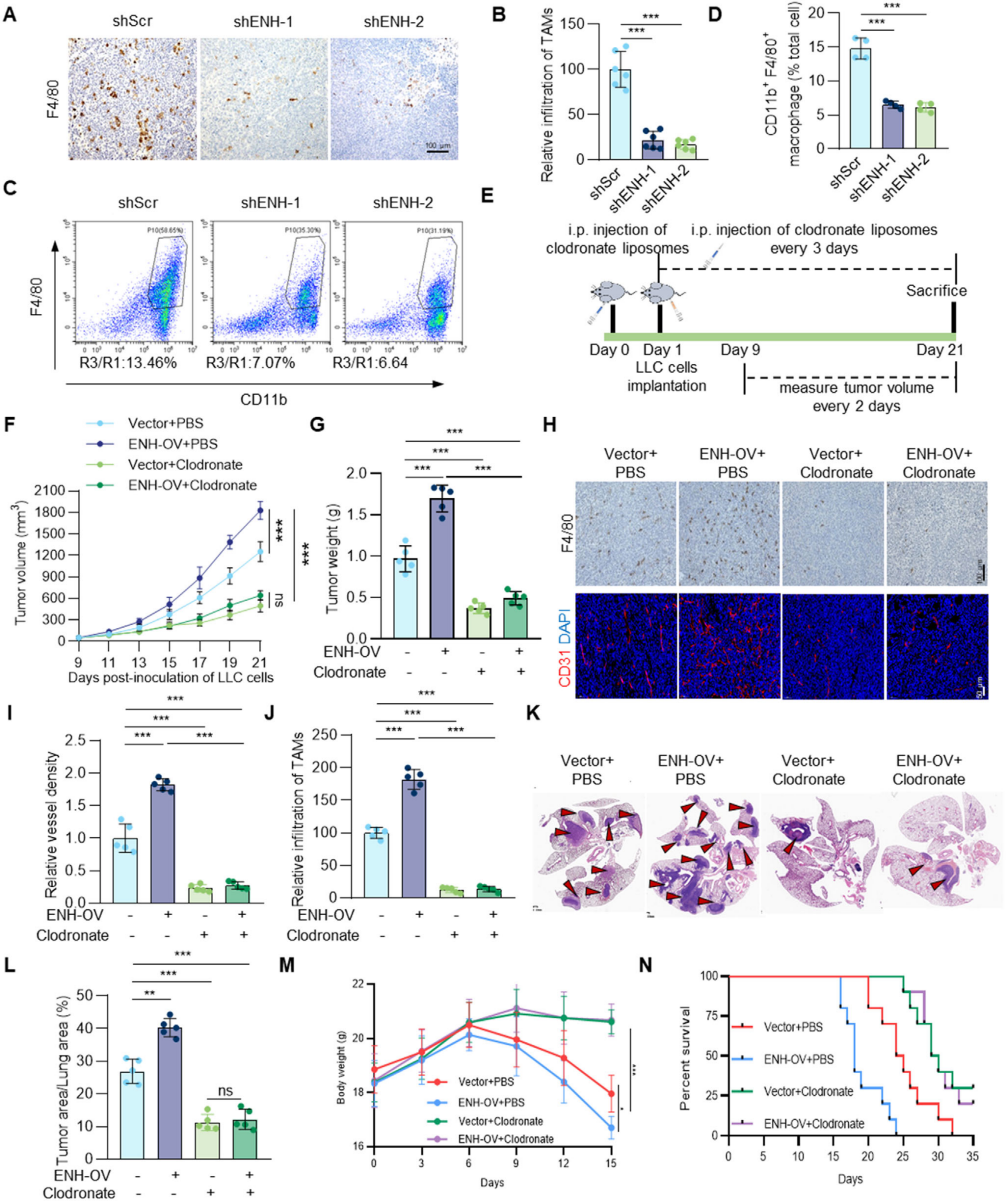
ENH‐induced tumor angiogenesis and growth are related to TAMs infiltration. A, B) IHC analysis of F4/80 positive cells in ENH knockdown or control LLC subcutaneous tumors sections was performed. The ratio of F4/80 positive cells was quantified and shown as a bar graph (*n* = 6). C, D) Representative flow cytometric plots of tumor‐infiltrating CD45^+^CD11b^+^F4/80^+^ macrophages in LLC subcutaneous tumors with or without ENH knockdown. The quantification of the proportion of infiltrating TAMs (CD45^+^CD11b^+^F4/80^+^/total cell, P10/R1) was shown as a bar graph (*n* = 4). E) Schematic for macrophage depletion in C57BL/6 mice implanted subcutaneously with LLC tumor cells. F) LLC‐Vector and LLC‐ENH‐OV tumor growth in mice treated with PBS or clodronate liposomes (*n* = 5). G) Weight of dissected tumors obtained from mice of indicated groups (*n* = 5). H) Representative images of IHC staining for F4/80 and IF staining for CD31 in LLC‐Vector and LLC‐ENH‐OV tumors sections treated with PBS or clodronate liposomes. I,J) Quantification of CD31^+^ vessels density and the ratio of F4/80 positive cells was shown. K) Representative images of HE staining of lung sections from orthotopic tumor‐bearing mice of indicated groups. Red arrows represent the tumors. L) The quantification of tumor burden of lung sections from indicated groups. M) The changes in body weight of orthotopic tumor‐bearing mice during the period of the experiment. N) Survival curve of orthotopic tumor‐bearing mice in indicated groups.

To investigate whether TAMs mediate ENH‐induced tumor growth and angiogenesis, we detected the content of TAMs using pan‐macrophage marker F4/80 in the subcutaneous tumor tissues of ENH knockdown and control groups. The detection was carried out when the tumor diameter in either group of mice approached 2 cm. We found that ENH knockdown significantly decreased TAMs content by ≈80% (**Figure** **2**A,B). Subcutaneous tumors were also harvested for flow cytometry analysis to compare immune cell infiltration. Consistent with the IHC staining results, flow cytometry analysis also showed a significant reduction of TAMs (CD45^+^/CD11b^+^/F4/80^+^) in ENH‐knockdown tumors compared to controls (Figure S7A, Supporting Information; Figure 2C,D). We then used clodronate liposomes to remove TAMs from the tumor tissue and confirmed the removal by F4/80 staining (Figure 2H). As shown in Figure 2E–J, ENH overexpression significantly promoted tumor growth and increased the levels of MVD and TAMs in tumors. Depletion of TAMs significantly attenuated ENH‐induced tumor growth and angiogenesis. Furthermore, an orthotopic xenograft mouse model was used to evaluate the role of ENH in driving tumor growth. Similar to the subcutaneous xenograft model, ENH overexpression significantly promoted orthotopic tumor progression, which was suppressed by TAMs depletion (Figure 2K,L). In addition, the body weight of control orthotopic tumor‐bearing mice declined markedly. ENH overexpression hastened the weight loss, which was restrained after TAM clearance (Figure 2M). Survival results showed that at 32 days, all control group mice died; ENH overexpression further shortened mice survival, whereas TAMs clearance significantly prolonged it (Figure 2N). By exploring the scRNA seq‐data in the TISCH database, we found that VEGFA was predominantly expressed in monocyte/macrophage populations (Figure S8A, Supporting Information). Further, data from GSE117570 showed that TAMs accounted for a large proportion of NSCLC tissues and confirmed the high expression of VEGFA in TAMs (Figure S8B–D, Supporting Information), implying that ENH participated in VEGF pathway by recruiting TAMs. All these results indicate that ENH drives LUAD tumor growth and angiogenesis by recruiting TAMs.

### 2.3 ENH Recruits TAMs in a CCL5‐Dependent Manner

**Figure 3:**
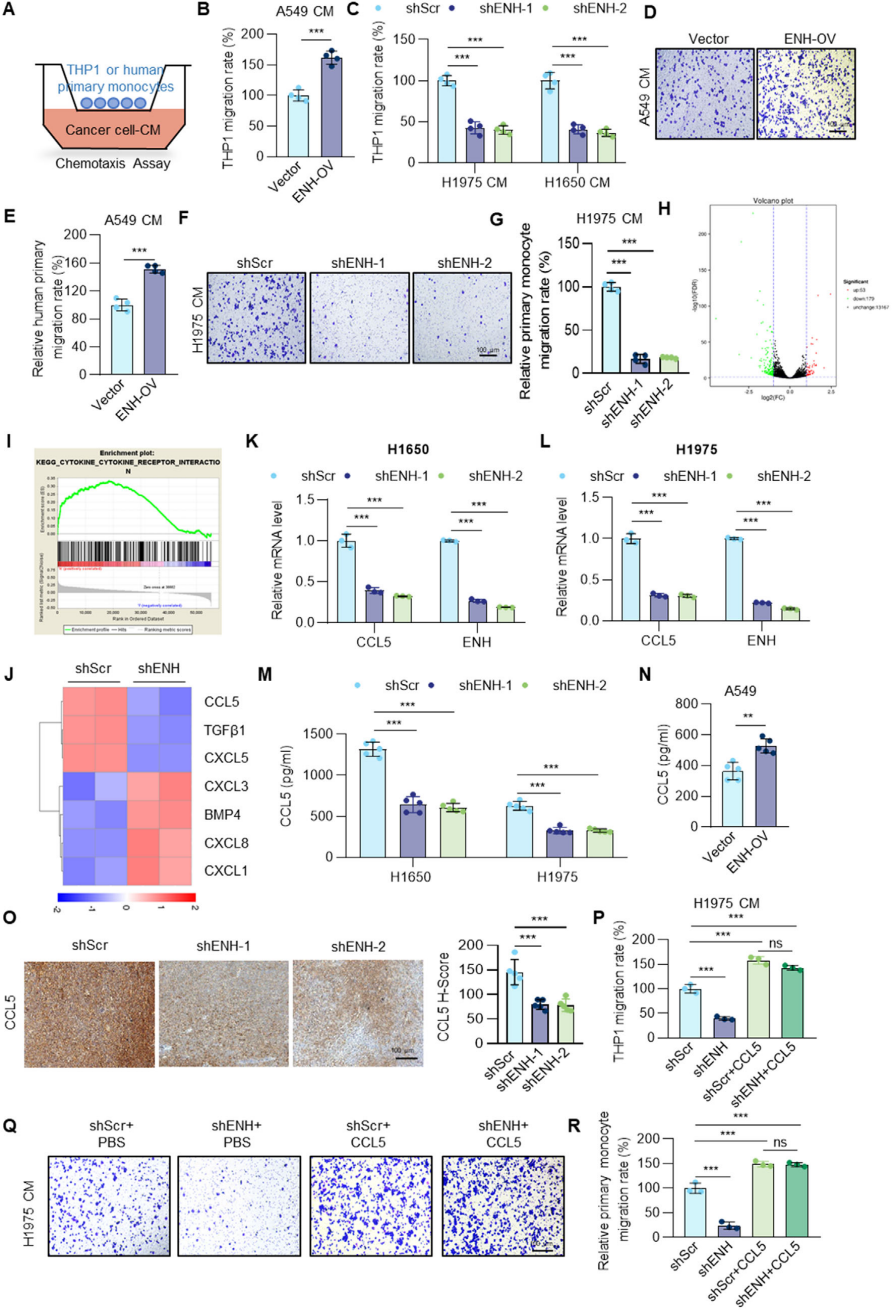
ENH recruits macrophages via CCL5. A) Scheme of the method for THP1 and human primary monocytes chemotaxis assay using CM of LUAD cells. B) The bar graph showed the number of migrated THP1 cells induced by CM isolated from ENH‐overexpressing A549 cells, represented as a relative percentage to the control (*n* = 4). C) The bar graph showed the number of migrated THP1 cells induced by CM isolated from ENH knockdown H1975 and H1650 cells, represented as a relative percentage to the control (*n* = 4). D, E) Representative images and a bar graph depicted the quantity of migrated human primary monocytes stimulated by CM isolated from ENH‐overexpressing A549 cells. The results were presented as a relative percentage compared to the control group (*n* = 4). F. G) Representative images and a bar graph depicted the quantity of migrated human primary monocytes stimulated by CM isolated from ENH knockdown H1975 cells. The results were presented as a relative percentage compared to the control group (*n* = 4). H) RNA sequencing of A549 cells with or without ENH knockdown. The volcano plot displayed the DEGs between the control and ENH knockdown groups. I) GSEA revealed that ENH expression mainly affected cytokine–cytokine receptor interaction pathway. J) Heatmap showing the expression of cytokines that are significantly altered in ENH knockdown A549 cells derived from RNA‐seq data. K, L) qPCR analysis was conducted to verify the downregulation of CCL5 mRNA levels in ENH knockdown H1975 and H1650 cells (*n* = 3). M) ELISA was conducted to verify the downregulation of CCL5 protein levels in ENH knockdown H1975 and H1650 cells (*n* = 5). N) ELISA was conducted to verify the upregulation of CCL5 protein level in ENH overexpression A549 cells (*n* = 5). O) Representative images of IHC staining for CCL5 in ENH knockdown or control LLC subcutaneous tumor sections. Statistical analysis of IHC results of CCL5 expression was shown as a bar graph (*n* = 5). P) CM collected from ENH knockdown cells added with or without rCCL5 protein was used as chemoattractants in THP1 chemotaxis assay. The bar graph showed the number of migrated THP1 cells, represented as a relative percentage to the control (*n* = 3). Q, R) CM collected from ENH knockdown cells added with or without rCCL5 protein was used as chemoattractants in human primary monocytes chemotaxis assay. Representative images and a bar graph depicted the quantity of migrated human primary monocytes. The results were presented as a relative percentage compared to the control group (*n* = 3).

At the tumor site, monocytes are recruited and then differentiated into TAMs. Therefore, we investigated the functional importance of ENH in regulating monocyte recruitment using the human monocyte cell line THP1 and human primary monocytes (**Figure** **3**A). Transwell assays showed that CM from ENH‐overexpressing LUAD cells enhanced monocyte recruitment compared to controls (Figure 3B,D,E). In contrast, CM from ENH‐knockdown cells inhibited the chemotaxis of monocytes (Figure 3C,F,G). To further determine how ENH regulates monocyte‐derived TAM infiltration, we implemented a transcriptome analysis using ENH stable knockdown and control A549 cells. Differential expression analysis showed that the expression levels of a total of 232 genes were significantly altered upon ENH knockdown (53 upregulated and 179 downregulated) (Figure 3H). It is well known that TAMs recruitment in tumors is mediated by cytokines, chemokines, and growth factors. Interestingly, the cytokine‐cytokine receptor interaction pathway was one of the largest enriched gene set clusters in ENH‐high LUAD cancer samples (Figure 3I).

We analyzed the differences in cytokine expression and found that only seven were significantly downregulated in ENH‐knockdown cells (Figure 3J). Among them, CCL5 is a major cytokine that has been shown in a number of studies to be critical for the attraction of TAMs.^[^ ^35^, ^36^ ^]^ On this basis, we focused on the role of CCL5 in ENH‐induced TAMs recruitment. We first confirmed the reduction of CCL5 mRNA in ENH‐knockdown cell lines (Figure 3K,L). Besides, ELISA analysis revealed that ENH knockdown decreased CCL5 protein secretion, whereas ENH overexpression increased it (Figure 3M,N). IHC staining further showed that CCL5 expression was decreased in ENH‐knockdown tumor tissues (Figure 3O). To investigate the association of ENH and CCL5 in patients with LUAD, we analyzed clinical data obtained from the GEO database, which confirmed that CCL5 mRNA levels were significantly and positively correlated with ENH mRNA levels (Figure S9A, Supporting Information). Additionally, qPCR and IHC assays showed a positive correlation between ENH expression and CCL5 expression in human LUAD specimens (Figure S9B–D, Supporting Information). To substantiate that ENH‐induced macrophage recruitment was mediated by CCL5, we used recombinant CCL5 (rCCL5) or CCL5 neutralizing antibodies (antiCCL5) to alter the concentration of CCL5 in CM used for chemotaxis assays. As shown in Figure S9E,F (Supporting Information), exogenous CCL5 addition enhanced monocyte recruitment, while blockade of CCL5 inhibited the chemotaxis of monocytes. Indeed, the addition of rCCL5 completely restored the chemotactic ability of ENH knockdown CM to monocytes (Figure 3P–R). Overall, these findings confirmed that ENH promotes TAMs recruitment in LUAD by increasing CCL5 levels.

### 2.4 ENH‐Induced Tumor Angiogenesis and Growth Depend on CCL5‐mediated TAMs Recruitment

To evaluate the clinical relevance of CCL5 expression in LUAD, we performed IHC on 15 paired tumors and adjacent normal tissues. The results confirmed upregulated CCL5 in LUAD tissues (Figure S10A,B, Supporting Information). CCL5 levels were also significantly higher in the serum of patients with LUAD compared to healthy controls (Figure S10C, Supporting Information). Notably, elevated CCL5 levels were positively associated with the malignant progression of patients with LUAD in terms of overall survival and relapse‐free survival (Figure S10D,E, Supporting Information). The relationship between CCL5 and TAMs infiltration in LUAD clinical samples was further investigated. Online database analysis showed that CCL5 expression was strongly positively correlated with both macrophage/monocyte markers and TAMs/monocyte infiltration in the tissues of patients with LUAD (Figure S10F,G, Supporting Information). Additionally, CCL5 expression was positively correlated with PECAM1 expression (Figure S10H,I, Supporting Information). These findings suggest that CCL5 is involved in TAMs recruitment and angiogenesis, contributing to the malignant progression of LUAD.

**Figure 4:**
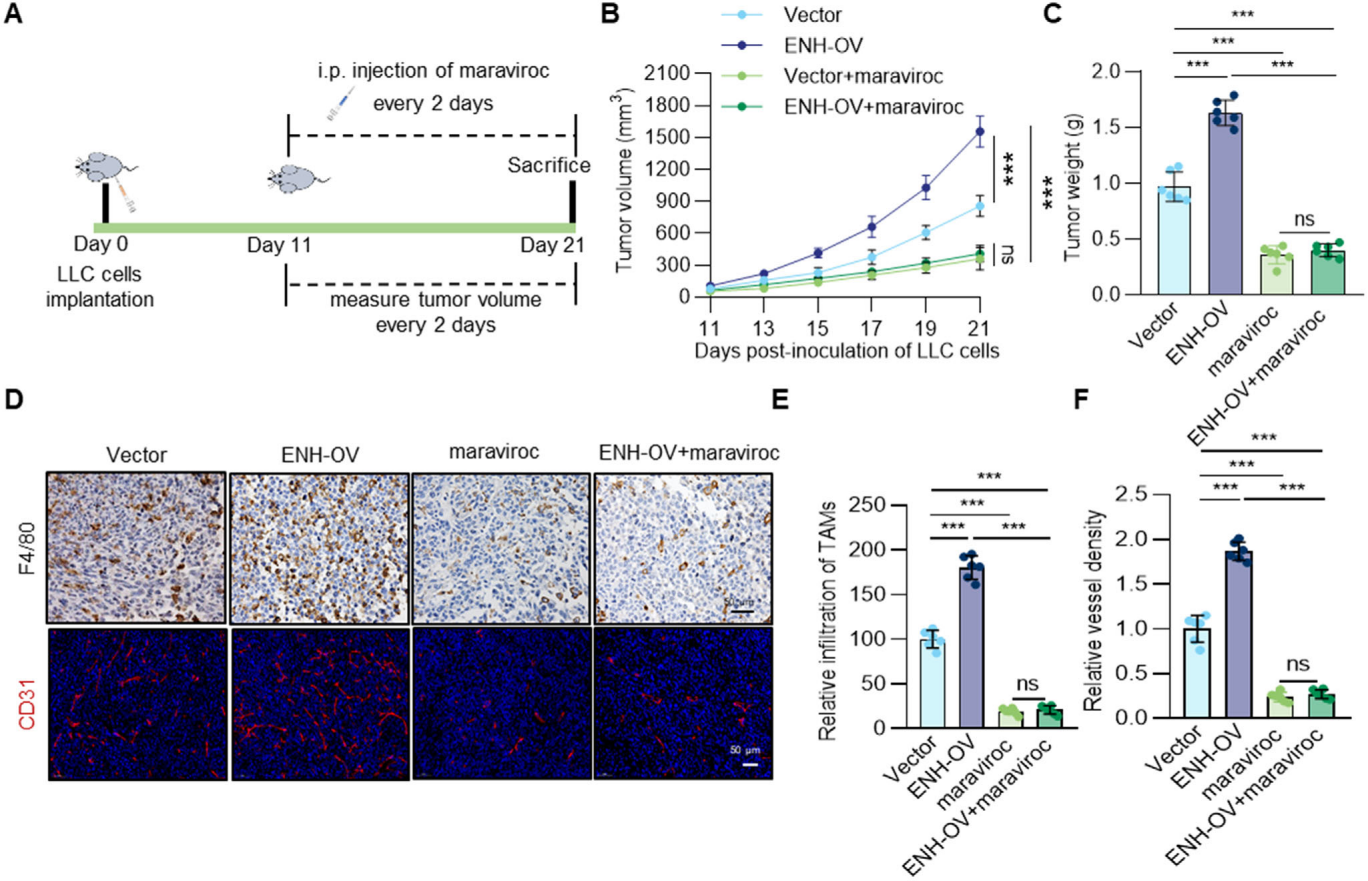
CCL5 is essential for ENH‐induced TAMs recruitment, tumor growth, and angiogenesis. A)Schematic for CCL5 signaling blocking in C57BL/6 mice implanted subcutaneously with LLC tumor cells. B) LLC‐Vector and LLC‐ENH‐OV tumor growth in mice treated with or without maraviroc (*n* = 6). C) Weight of dissected tumors obtained from mice of indicated groups (*n* = 6). D) Representative images of IF staining for CD31 and IHC staining for F4/80 in LLC‐Vector and LLC‐ENH‐OV tumors sections treated with or without maraviroc. E,F) The ratio of F4/80 positive cells and CD31^+^ vessel density was quantified and shown as a bar graph (*n* = 6).

To directly investigate the role of CCL5 in angiogenesis, we conducted in vitro studies with rCCL5, which showed that rCCL5 had no impact on endothelial cell migration, proliferation, or tube formation compared to the control, suggesting that CCL5 does not directly induce angiogenesis (Figure S11A–E, Supporting Information). Therefore, we hypothesized that CCL5 indirectly promotes angiogenesis and malignant progression by recruiting TAMs. To confirm the hypothesis, we constructed a xenograft tumor model and injected the mice with maraviroc, a CCR5 antagonist, thus blocking the CCL5‐CCR5 axis. Maraviroc markedly inhibited subcutaneous tumor growth and completely abolished the accelerated tumor growth caused by ENH overexpression (**Figure** **4**A–C). Furthermore, maraviroc treatment reduced ENH‐induced TAMs recruitment and angiogenesis (Figure 4D–F). We concluded that ENH upregulates CCL5, leading to an accumulation of TAMs in LUAD, which ultimately promotes tumor angiogenesis and growth.

### 2.5 ENH Promotes M2‐Like Polarization of TAMs Through CCL5‐Induced STAT3 Activation

**Figure 5:**
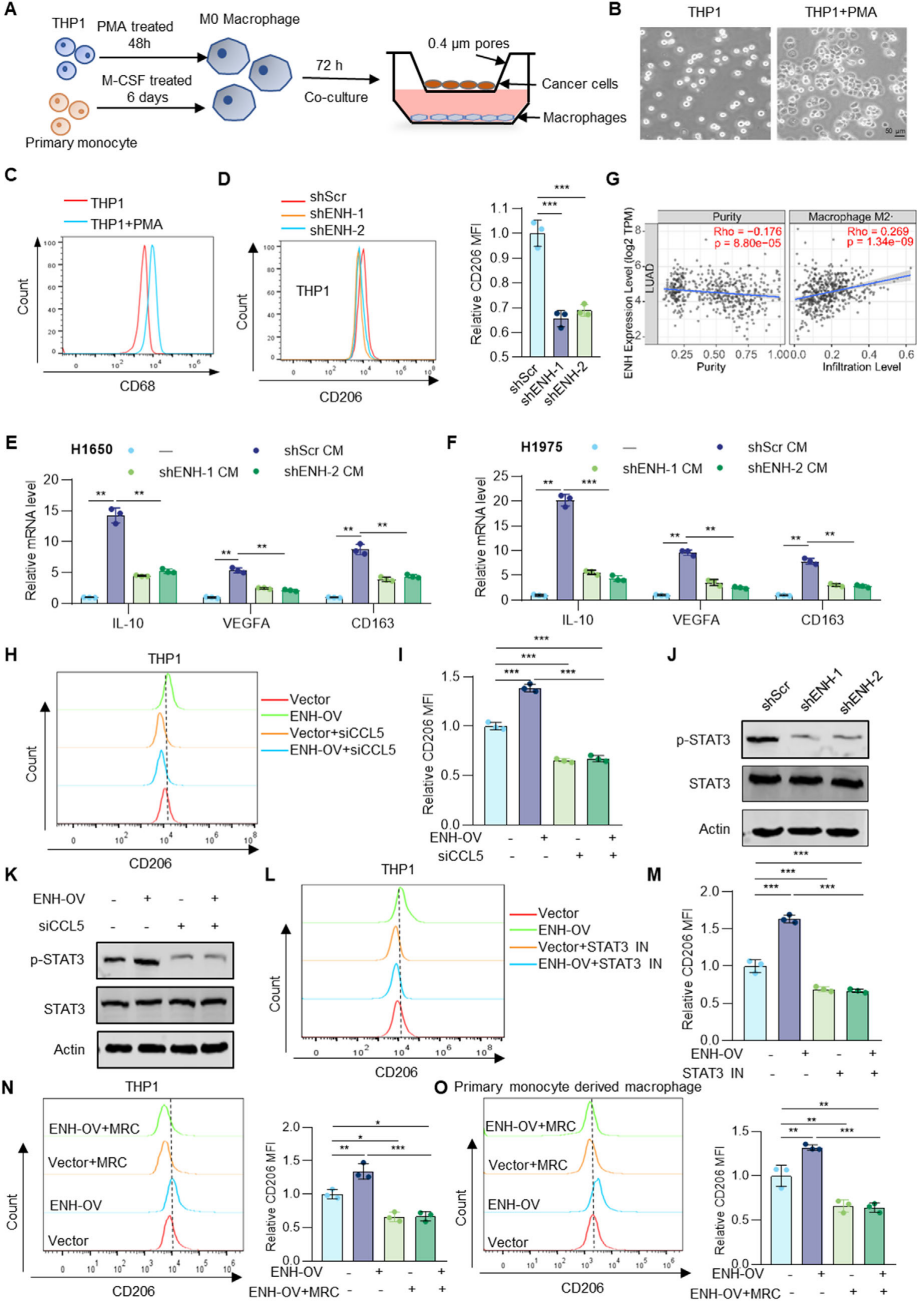
ENH activates macrophage STAT3 signaling through CCL5 to promote M2 polarization. A) Schematic model of the co‐culture system of THP1 and human primary monocytes‐drived macrophages with LUAD cells. B) Representative images of THP‐1 cells before and after PMA treatment. C) Flow cytometric analysis of CD68 expression in PMA‐treated or untreated THP‐1 cells. D) Flow cytometric analysis of CD206 expression in THP1‐drived macrophages co‐cultured with ENH knockdown LUAD cells. The mean fluorescence intensities (MFI) value of each group was represented in the histogram (*n* = 3). E,F) The mRNA levels of M2 markers in THP1‐drived macrophages co‐cultured with ENH knockdown LUAD cells were measured by qPCR (n = 3). G) The correlation of ENH with M2 macrophage infiltration in LUAD was analyzed using the TIMER 2.0 database. H, I) Flow cytometric analysis of CD206 expression in THP1‐drived macrophages co‐cultured with ENH overexpression and CCL5 knockdown LUAD cells. The MFI value of each group was represented in histogram (*n* = 3). J) Western blot analysis of STAT3/p‐STAT3 levels in THP1‐drived macrophages co‐cultured with ENH knockdown LUAD cells. K) Western blot analysis of STAT3/p‐STAT3 levels in THP1‐drived macrophages co‐cultured with ENH overexpression and CCL5 knockdown LUAD cells. L,M) Flow cytometric analysis of CD206 expression in THP1‐drived macrophages co‐cultured with ENH overexpression LUAD cells treated with or without STAT3 inhibitor (STAT3‐IN). The MFI value of each group was represented in the histogram (*n* = 3). N,O) Flow cytometric analysis of CD206 expression in THP1‐ and human primary monocytes‐derived macrophages co‐cultured with ENH overexpression LUAD cells treated with or without maraviroc (MRC). The MFI value of each group was represented in histogram (*n* = 3).

We also examined whether ENH affects macrophage polarization in LUAD. THP‐1 cells were induced into M0 macrophages by PMA, and human primary monocytes were differentiated into M0 macrophages by M‐CSF. Then, these M0 macrophages were co‐cultured with LUAD cells (**Figure** **5**A–C). The results showed that compared to the control, cells co‐cultured with ENH‐knockdown LUAD cells exhibited lower expression of M2 markers and a CD206^low^ phenotype (Figure 5D–F; Figure S12A, Supporting Information). In line with this, bioinformatics analysis through the TIMER database revealed a positive association between ENH expression and M2 TAMs infiltration (Figure 5G).

To determine whether ENH affects M2 polarization through CCL5, we knocked down CCL5 in ENH‐overexpressing LUAD cells before co‐culture. Flow cytometry results showed that overexpression of ENH increased the proportion of M2 macrophages, while the knockdown of CCL5 completely abrogated this appearance (Figure 5H,I; Figure S12B, Supporting Information). M2‐polarized macrophages have been reported to have a higher angiogenic potential compared to other subsets.^[^ ^37^ ^]^ We then performed tube formation and migration assays using endothelial cultured by CM collected from co‐cultures of LUAD cells and macrophages. The results showed that the CM collected from the co‐culture system containing ENH‐overexpressing cells enhanced tube formation and migration ability of endothelial, which was dramatically attenuated by CCL5 knockdown (Figure S12C–E, Supporting Information). These findings suggest that, besides migration, ENH also promotes M2‐like polarization of macrophages in a CCL5‐dependent manner, thereby promoting angiogenesis.

Previous studies have reported that CCL5 induces STAT3 activation, a key driver of M2 polarization.^[^ ^38^, ^39^ ^]^ Therefore, we investigated whether ENH‐induced M2 polarization was mediated by STAT3 activation. Western blot analysis showed decreased STAT3 activation in macrophages co‐cultured with ENH‐knockdown LUAD cells compared to controls (Figure 5J). Additionally, CCL5 knockdown abolished ENH overexpression‐induced STAT3 activation (Figure 5K). Moreover, treated macrophages with STAT3 inhibitor also effectively blocked M2 polarization induced by ENH‐overexpressing tumor cells (Figure 5L,M; Figure S12F, Supporting Information). Using maraviroc to block the CCL5‐CCR5 signaling pathway also notably reduced the macrophage M2 polarization triggered by ENH‐overexpressing CM (Figure 5N,O). Combined, these results indicate that ENH caused poor prognosis in patients with LUAD also relies on the functional reprogramming of macrophages in a CCL5‐dependent manner.

### 2.6 ENH Promotes LUAD Malignant Progression by Retaining Nuclear YAP and Thus Increasing CCL5 Expression

To investigate how ENH regulates CCL5 expression, we performed KEGG enrichment analyses using DEGs identified by transcriptome analysis between ENH‐knockdown and control cells. The analysis showed that the DEGs were significantly enriched in the Hippo signaling pathway (Figure S13A, Supporting Information). Dysregulation of the Hippo pathway has been shown to be involved in cancer progression. YAP is the effector protein of the Hippo pathway that interacts with transcription factors such as TEAD through nuclear translocation to regulate the gene transcription program. Through qPCR and ELISA analysis, we confirmed that YAP overexpression dramatically elevated the mRNA and protein content of CCL5 (Figure S14A–C, Supporting Information). Conversely, genetic and pharmacological inhibition of YAP significantly reduced the mRNA and protein expression of CCL5 (Figure S14D–G, Supporting Information).

**Figure 6:**
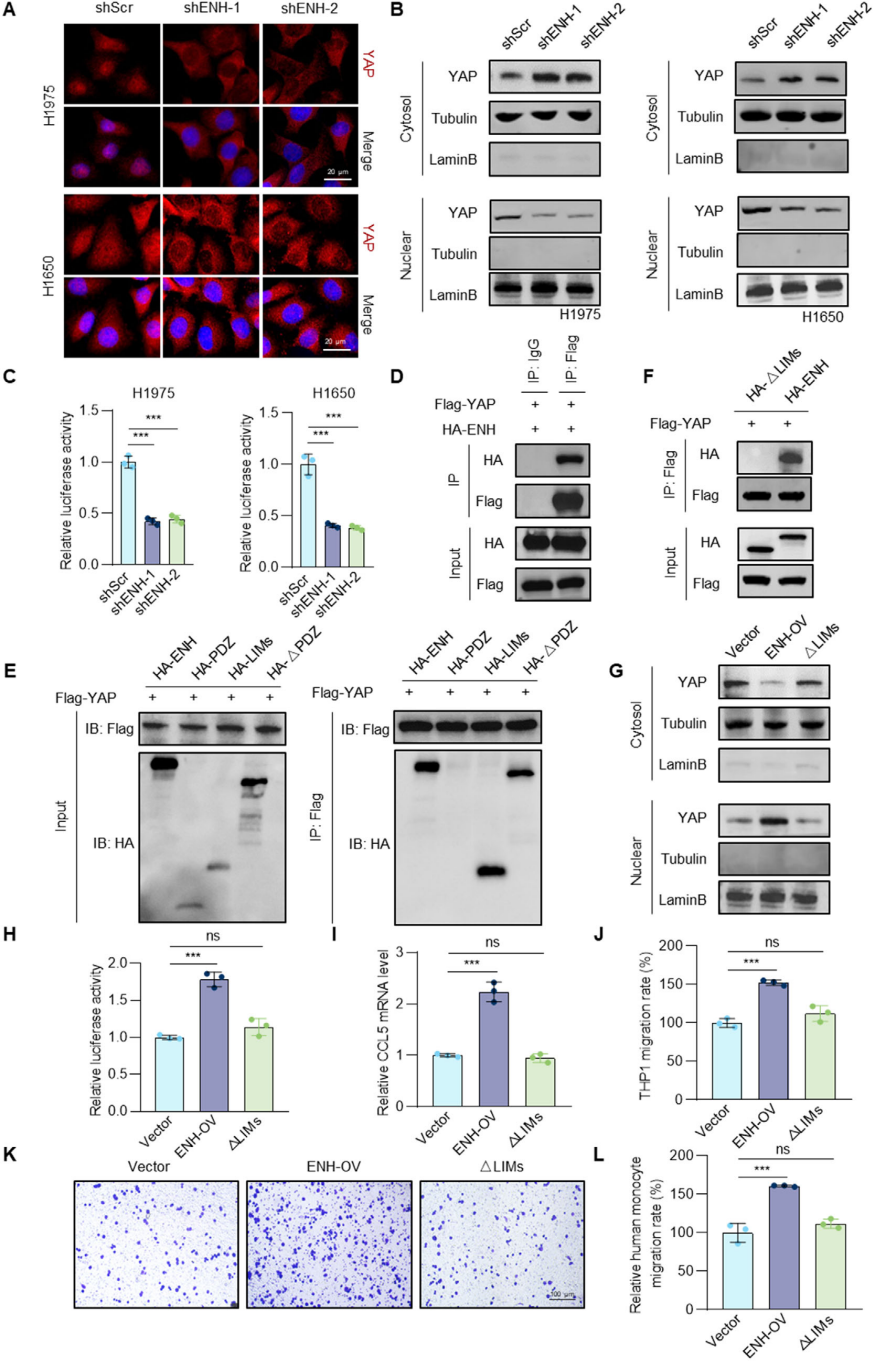
ENH interacts with YAP to promote its nuclear translocation. A) Representative images of IF staining for YAP in ENH knockdown H1975 and H1650 cells. B) Western blot analysis of YAP cellular localization changes in ENH knockdown H1975 and H1650 cells. C) YAP transcriptional activity in H1650 and H1975 cells expressing the 8× GTIIC luciferase reporter. Cells were silenced with ENH shRNA (*n* = 3). D) CO‐IP analysis of the interaction between ENH and YAP in HEK293T cells. E) Mapping ENH fragments that interacted with YAP. HEK293T cells were co‐transfected with FLAG–YAP and ENH truncated fragment (HA‐ENH, 1–596 amino acids; LIMs, 418–596 amino acids; △PDZ, 85–596 amino acids; PDZ, 1–85 amino acids) for immunoprecipitation assays. F) CO‐IP analysis of the interaction between YAP and ENH LIM domain deletion mutant (△LIMs) in HEK293T cells. G) Western blot analysis of YAP cellular localization changes in A549 cells overexpressing ENH full‐length or LIM‐domain deletion mutant. H) YAP transcriptional activity was detected in A549 cells overexpressing ENH full‐length or LIM‐domain deletion mutant (*n* = 3). I) qPCR analysis of CCL5 mRNA levels in A549 cells overexpressing ENH full‐length or LIM‐domain deletion mutant (*n* = 3). J) CM collected from A549 cells overexpressing ENH full‐length or LIM‐domain deletion mutant was used as chemoattractant in THP1 chemotaxis assay. The bar graph showed the number of migrated THP1 cells, represented as a relative percentage to the control (*n* = 3). K, L) Representative images and a bar graph depicted the quantity of migrated human primary monocytes stimulated by CM isolated from ENH or LIM‐domain deletion mutant overexpression A549 cells. The results were presented as a relative percentage compared to the control group (*n* = 3).

Next, we determined whether ENH directly regulated YAP expression. The results showed that neither mRNA nor protein levels of YAP were altered upon ENH knockdown (Figure S14H,I, Supporting Information). Therefore, we hypothesized that ENH regulated CCL5 expression by modulating the nuclear translocation of YAP. We examined the distribution of YAP in cells with or without ENH knockdown. IF staining revealed that ENH knockdown attenuated nuclear YAP accumulation (**Figure** **6**A), and western blot analysis confirmed decreased nuclear YAP protein levels and significantly increased cytoplasmic YAP levels upon ENH depletion (Figure 6B). To assess whether ENH functionally regulated YAP, we used 8×GTIIC luciferase reporter to monitor YAP transcriptional activity and found that ENH knockdown significantly decreased luciferase reporter activity compared to the control (Figure 6C). Furthermore, CCL5 upregulation mediated by ENH overexpression was completely abrogated after YAP knockdown whereas YAP overexpression rebounded CCL5 levels in ENH‐knockdown cells (Figure S14J,K, Supporting Information). Chemotaxis assays showed that CM from YAP over‐expressing cells promoted monocyte chemotaxis, while CM from YAP‐knockdown cells dramatically inhibited monocyte chemotaxis, which was restored by the addition of CCL5 (Figure S14L–M, Supporting Information). Rescue experiments revealed that YAP knockdown inhibited the increase in monocyte chemotaxis induced by ENH overexpression, while YAP overexpression inhibited the decrease in monocyte chemotaxis induced by ENH knockdown (Figure S14N,O, Supporting Information). Altogether, these findings suggest that ENH stimulates CCL5 expression by inducing YAP nuclear translocation, which promotes TAMs infiltration, angiogenesis, and tumor progression.

### 2.7 ENH Promotes YAP Nuclear Accumulation By Interacting with it

LATS1‐2/‐induced serine 127 phosphorylation of YAP is critical for nucleocytoplasmic shuttling of YAP.^[^ ^40^ ^]^ However, ENH depletion did not affect pS127 YAP levels (Figure S14I, Supporting Information). Interestingly, we found that YAP is a potential ENH‐binding protein (Figure S13B,C, Supporting Information), then proceeded to examine whether ENH regulated YAP nuclear distribution by interacting directly with it. First, to study the association of ENH with YAP, we performed CO‐IP by ectopically expressing them in 293T cells. Protein pellets obtained from YAP precipitation using anti‐FLAG antibody contained the HA‐tagged ENH protein (Figure 6D). We further confirmed this interaction in A549 cells by performing CO‐IP assays using endogenous proteins (Figure S13D, Supporting Information). Next, we proceeded to map the interacting domains of ENH and YAP and found that the LIMs domain of ENH, rather than the PDZ domain, was responsible for binding (Figure 6E). We also demonstrated that ENH truncations lacking LIM domains (ΔLIMs) failed to interact with YAP (Figure 6F). Importantly, overexpression of wild‐type (WT) ENH enhanced the nuclear translocation of YAP whereas the ΔLIMs ENH construct did not have this effect (Figure 6G). Additionally, 8×GTIIC luciferase reporter activity was significantly increased in LUAD cells overexpressing WT ENH, whereas expression of ΔLIMs ENH constructs had no similar effect (Figure 6H). These results suggest that ENH regulates the cellular localization and activity of YAP dependent on the interaction. Next, we explored whether the ENH‐YAP interaction affects CCL5 expression. As expected, ΔLIMs ENH failed to upregulate CCL5 in LUAD cells (Figure 6I). Consistently, overexpression of WT ENH, not ΔLIMs ENH, enhanced the monocyte recruitment (Figure 6J–L). Overall, these findings indicate that the regulation of CCL5 expression by ENH is reliant on YAP‐ENH interactions.

### 2.8 ENH Promotes YAP Nuclear Translocation by Inducing its Binding to KPNA2

**Figure 7:**
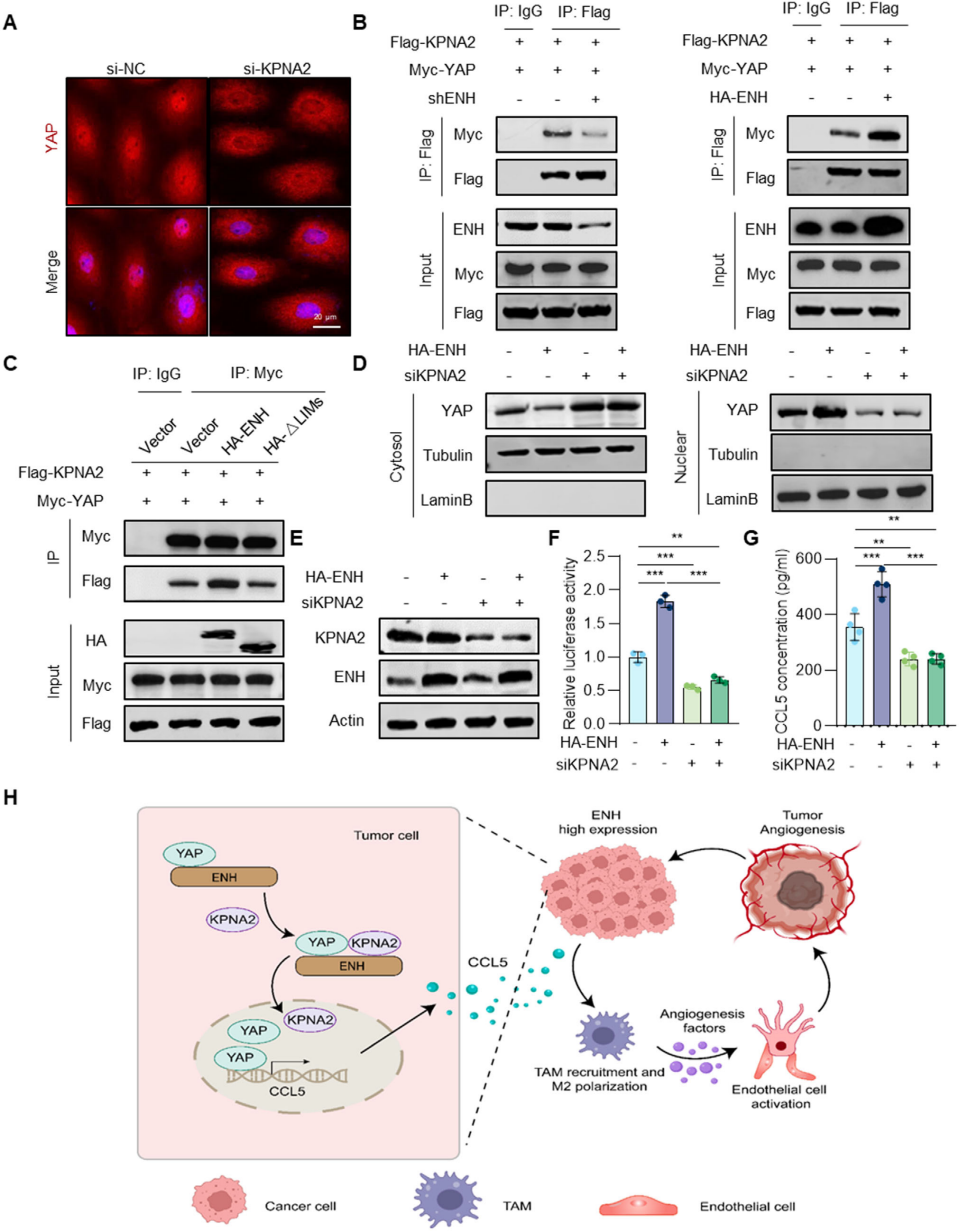
ENH interacts with YAP to induce its binding to KPNA2, followed by nuclear translocation. A) Representative images of IF staining for YAP in KPNA2 knockdown H1650 cells. B) CO‐IP analysis of the interaction between YAP and KPNA2 in H1650 cells with ENH knockdown or overexpression. C) CO‐IP analysis of the interaction between YAP and KPNA2 in H1650 cells overexpressing ENH full‐length or LIM‐domain deletion mutant. D) Western blot analysis of YAP cellular localization changes in ENH overexpression A549 cells with or without KPNA2 knockdown. E) Efficiency of ENH overexpression and KPNA2 knockdown in A549 cells was detected by western blot analysis. F) YAP transcriptional activity was detected in ENH overexpression A549 cells with or without KPNA2 knockdown (*n* = 3). G) ELISA analysis of CCL5 protein levels in ENH overexpression A549 cells with or without KPNA2 knockdown (*n* = 4). H) The schematic diagram shows the general process and mechanism of this study. ENH in tumor cells induces the binding of YAP to KPNA2, triggering YAP nucleus entry to up‐regulate CCL5 transcription, thus promoting tumor angiogenesis and growth by driving TAMs infiltration and M2 polarization.

Based on the size of YAP (55 kDa), it is imported into the nucleus of the cell by binding to the nuclear transporter proteins, specifically mediated by the importin α/β transport system.^[^ ^41^ ^]^ To identify the importins responsible for YAP nuclear translocation, we performed a screening using a double luciferase reporter system in the cell with siRNA knockdown of the indicated importin. We observed that importin α1 (KPNA2) knockdown most significantly attenuated the activity of the 8× GTIIC luciferase reporter (Figure S15A, Supporting Information). Furthermore, we confirmed that YAP binds to KPNA2 (Figure S15B, Supporting Information). IF staining showed that knockdown of KPNA2 dramatically inhibited YAP nuclear translocation in H1650 cells (**Figure** **7**A). These findings indicate that KPNA2 is the major importin responsible for transporting YAP into the nucleus.

Considering the properties of ENH, we hypothesized that it regulates YAP nuclear translocation by influencing the interaction between YAP and KPNA2. To validate this hypothesis, we examined whether ENH was essential for the binding of YAP to KPNA2. The CO‐IP results showed that ENH overexpression increased YAP association with KPNA2, whereas ENH knockdown attenuated their interaction (Figure 7B). Additionally, although ENH promoted the binding of YAP and KPNA2, ΔLIMs ENH did not, suggesting that the association of ENH with YAP is important for regulating the formation of YAP and KPNA2 complexes (Figure 7C). We, therefore, investigated whether ENH‐induced YAP nuclear import was KPNA2 dependent. We knocked down KPNA2 in ENH‐overexpression cells and found that the knockdown of KPNA2 completely inhibited the increased nuclear import of YAP in ENH‐overexpressing cells (Figure 7D). Furthermore, ENH overexpression‐induced increase in 8× GTIIC luciferase reporter activity and upregulation of CCL5 expression were also abrogated upon KPNA2 knockdown (Figure 7E–G). Compared with the control group, the CM of LUAD cells with KNPA2 knockdown significantly inhibited the recruitment of monocytes (Figure S15C,D, Supporting Information). These results suggest that the presence of ENH promotes the formation of a complex between YAP and KPNA2, thereby facilitating the nuclear retention and activation of YAP to transcribe CCL5 and recruit monocytes. (Figure 7H).

### 2.9 ENH Knockdown Enhances the Chemotherapeutic Efficacy of Cisplatin

Considering that TAM infiltration and angiogenesis contribute significantly to chemotherapy resistance, we investigated whether ENH inhibition could enhance the efficacy of chemotherapy in LUAD. We established an LLC subcutaneous xenograft mouse model using ENH‐knockdown cells. Mice were treated with cisplatin (4 mg kg^−1^) via intravenous injection every 3 days. The results showed that the combination of ENH knockdown and cisplatin significantly inhibited tumor progression compared to the single treatment group, as evidenced by changes in tumor volume, and tumor weight (Figure S16A–C, Supporting Information). These findings suggest that ENH could be an effective target for LUAD treatment.

## 3 Discussion

For many tumors, including LUAD, currently available therapies are ineffective, and patient prognosis remains poor.^[^ ^42^ ^]^ Tumor cells acquire interdependencies with other cells in the TME to promote tumor angiogenesis and immune tolerance, thus promoting tumor progression and refractoriness.^[^ ^43^, ^44^, ^45^ ^]^ Understanding how tumor cells interact with surrounding cells in the TEM during tumor development and then developing novel anti‐tumor drugs has been the focus of anti‐tumor therapy. This study revealed a novel mechanism underlying the interplay between tumor cells and the tumor stromal cells, as we showed that the scaffolding protein, ENH, in tumor cells promotes tumor angiogenesis and growth by recruiting and facilitating M2 polarization of TAMs, confirming that targeting ENH has a promising antitumor effect.

Increased ENH expression is associated with poor prognosis and treatment resistance in multiple tumors, such as lung and prostate cancer. To date, the predominant role of ENH in tumor progression has been thought to be based on tumor cell‐intrinsic mechanisms such as promoting proliferation and metastasis. Our study is the first to demonstrate that ENH acts as a potent inducer of angiogenesis, which in turn promotes LUAD progression in a tumor cell nonautonomous manner.

It is well known that tumor cells interact with endothelial cells through secretory proteins to enhance angiogenesis.^[^ ^46^, ^47^, ^48^ ^]^ However, we found the CM from ENH‐overexpressing or knockdown LUAD cells did not alter the angiogenic ability of endothelial cells, suggesting that ENH may promote angiogenesis by remodeling the composition of immune cells. TAMs are important mediators that promote tumor angiogenesis by secreting pro‐angiogenic factors and facilitating the degradation of the perivascular extracellular matrix.^[^ ^49^, ^50^ ^]^ Multiple oncology studies have confirmed that TAM counts are associated with MVD and that inhibiting TAMs recruitment significantly delays tumor progression.^[^ ^51^, ^52^ ^]^ We found that ENH expression was positively correlated with multiple macrophage markers in LUAD specimens and that ENH overexpression in LUAD cells led to TAMs recruitment in vivo and in vitro. Furthermore, depletion of TAMs using clodronate liposomes completely blocked ENH‐induced tumor angiogenesis and growth. Collectively, these findings revealed a novel function of ENH to mediate tumor‐stromal cell communication by recruiting TAMs to facilitate LUAD angiogenesis and growth.

We also observed that ENH induces polarization of macrophages to an M2 phenotype, and CM from co‐culture of macrophages and ENH‐overexpressing LUAD cells enhances the angiogenic ability of endothelial. The results indicate that, in addition to recruitment, ENH also drives tumor progression in part by promoting the formation of a pro‐angiogenic phenotype in macrophages. Notably, in this study, we focused on the role of ENH‐induced TAMs recruitment and polarization in angiogenesis; however, in addition to angiogenesis, TAMs have also been shown to contribute to many steps in tumor progression.^[^ ^53^, ^54^ ^]^ Substantial evidence suggests TAMs directly mediate tumor cell proliferation, invasion, and motility. TAMs also promote immunosuppression by inducing T‐cell dysfunction.^[^ ^55^ ^]^ Thus, ENH‐mediated TAMs recruitment and polarization probably also play critical roles in other steps of tumor progression, a hypothesis that requires future studies.

Previous studies have identified chemokines such as CCL‐2 and CSF‐1 as major regulators of macrophage chemotaxis in tumors.^[^ ^56^ ^]^ In contrast, our study found that ENH affects TAM recruitment, as well as subsequent tumor progression, by triggering CCL5 expression. Blocking CCL5 signaling significantly inhibits ENH‐induced TAMs recruitment, neovascularization, and tumor growth. CCL5, also known as RANTES, is expressed by a variety of cells, including T cells, macrophages, platelets, and tumor cells, and it has been shown that CCL5 contributes to tumor growth, metastasis, and immune escape.^[^ ^57^, ^58^, ^59^ ^]^ Several studies have reported that CCL5 exerts its tumor‐progressive effects mainly through the recruitment of TAMs.^[^ ^60^, ^61^, ^62^, ^63^ ^]^ CCL5 also plays a role in macrophage programming; however, this role is controversial, as some studies have shown that CCL5 induces M2 polarization, while others reveal that CCL5 promotes M1 polarization.^[^ ^64^, ^65^ ^]^ We found that CM of ENH‐overexpressing cells activates STAT3 signaling in macrophages via CCL5, promoting M2 polarization. This result provides supportive evidence for a model of the pro‐M2 polarizing effect of CCL5. Our study also observed an increased expression of CCL5 in patients with LUAD patients and its correlation with poor prognosis. Monitoring CCL5 level and targeting CCL5 offers the possibility of accurate diagnosis and treatment of LUAD.

In this study, we found that ENH exerts tumor‐promoting effects, which is attributed to its ability to regulate YAP nuclear translocation. In LUAD, YAP is considered to play an important role in promoting tumor cell proliferation.^[^ ^66^ ^]^ However, our study showed that the pro‐carcinogenic effect of ENH‐YAP is not cell‐autonomous or proliferation‐dependent; rather, ENH‐activated YAP promotes CCL5 transcription, which in turn promotes TAMs recruitment and M2 polarization. In line with our findings, many studies have emphasized that YAP facilitates tumor progression by mediating heterologous communication between tumor cells and immune cells via paracrine‐acting factors.^[^ ^67^, ^68^, ^69^ ^]^ The macrophage recruitment, as well as the M2 polarization‐promoting function of YAP in cancer, has also been well established.^[^ ^70^, ^71^, ^72^ ^]^ The reason why ENH‐induced YAP activation does not affect cell proliferation may be that when ENH is knocked down or overexpressed, certain unknown molecules are also altered. These changes could result in the eventual equilibrium in the signals regulating tumor cell proliferation.

Our study found that ENH regulates YAP nuclear translocation in a Hippo pathway‐independent manner by directly binding to YAP. The nucleocytoplasmic distribution of YAP is believed to be mediated by the classical importin α/β heterodimer complex.^[^ ^73^, ^74^ ^]^ Factors affecting the interaction of YAP with importin molecules influence the nuclear import of YAP. For example, it has been reported that circRILPL1 promotes the binding of YAP and IPO7 thereby promoting YAP nuclear translocation.^[^ ^75^ ^]^ In addition, MST4 disrupts the interaction of YAP with importins by mediating the phosphorylation of YAP Thr83, thereby inhibiting its nuclear import.^[^ ^76^ ^]^ Our study also found that ENH promotes the formation of the KPNA2‐YAP complex, thereby enhancing nuclear translocation and YAP transcriptional activity. We also found that the interaction between ENH and YAP is necessary for YAP‐KPNA2 complex formation. As we overexpressed the LIM‐domain deletion ENH mutation disrupting the interaction between ENH and YAP, it failed to enhance YAP binding to KPNA2, and thus cannot enhance YAP nuclear translocation. However, the detailed molecular mechanisms by which ENH regulates the formation of the YAP‐KPNA2 complex require further studies, possibly involving post‐translation modifications.

Inhibition of angiogenesis has always been an important strategy in the clinical treatment of solid tumors. However, the use of current antiangiogenic drugs has not been as successful as expected, providing only minimal benefits to patient survival.^[^ ^77^, ^78^ ^]^ Approaches targeting TAMs recruitment and polarization are very promising new strategies for inhibiting angiogenesis. These strategies not only inhibit angiogenesis but also enhance the efficacy of chemotherapy and immunotherapy by remodeling the TME.^[^ ^79^, ^80^ ^]^ Our study demonstrates that targeting the ENH/CCL5 axis shows strong antiangiogenic and anti‐tumor activity by suppressing TAMs recruitment and M2 polarization, along with significantly improving chemotherapy outcomes. Further exploration of the potential for targeting ENH‐CCL5 to sensitize tumors to immunotherapy is warranted.

## 4 Conclusion

Overall, we demonstrated that ENH regulates the crosstalk between LUAD cells and macrophages through the KPNA2/YAP/CCL5 signaling axis. This interaction promotes macrophage recruitment and M2 polarization, contributing to tumor angiogenesis and growth. This new knowledge about the complex interactions between tumor cells and immune cells may provide better strategies for treating LUAD in the future.

## 5 Experimental Section

### Human Sample Collection

Forty samples with only LUAD tumor tissues and 15 samples containing tumor and adjacent normal tissues were obtained from the Fourth Affiliated Hospital, College of Medicine, Zhejiang University (Yiwu, China), and confirmed by pathological diagnosis. Serum samples were collected from 33 patients with LUAD and 21 healthy volunteers. Written informed consent was acquired from all patients prior to the study. This study was approved by the Research Ethics Committee of the Fourth Affiliated Hospital, College of Medicine, Zhejiang University (Yiwu, China, No. K2023161) and adhered to the Declaration of Helsinki principles.

### Double Luciferase Reporter Assay

LUAD cells were co‐transfected with 8×GTIIC‐luciferase reporter plasmids (1 µg per well) and Renilla luciferase plasmids (10 ng per well) in 24‐well plates to monitor the transcriptional activity of YAP. After 24 h, cells were lysed using a frozen lysis buffer. We centrifuged the lysate and placed 20 µL of supernatant in a 96‐well plate for detection. The luciferase activities of the reporter and Renilla were measured using the Dual‐Luciferase Reporter Assay System (Yeasen, Shanghai, China) according to the manufacturer's instructions. The reporter's firefly luciferase activity was quantified after normalization with Renilla luciferase activity.

### Monocyte Chemotaxis Assay

THP1 chemotaxis assays were performed using 5 µm pore size in 24‐well Transwell chambers (Coring, Armonk, NY, USA). 100 µL of 5 × 10^5^ THP‐1 monocytes resuspended in serum‐free RPMI 1640 medium containing 0.5% bovine serum albumin were seeded into the upper chambers. The lower chambers contained 600 µL mixed medium (50% CM from LUAD cells + 50% total medium). After 3 h incubation, cells that migrated to the lower chambers were collected and counted using a cell counter and flow cytometer.

Human peripheral blood mononuclear cells (PBMCs) were obtained from healthy subjects by density gradient centrifugation. Subsequently, monocytes were sorted and purified by flow cytometric sorting (labeled with anti‐CD14‐PE). The isolated monocytes were used for further chemotaxis experiments. In terms of the main steps, it is quite similar to the situation of THP1. However, there are certain differences. The migration of primary monocytes takes 24 h. When observing the results, the upper chamber is collected and then fixed with methanol, while the non‐migrated cells are removed. After that, the migrated and invaded cells are stained with crystal violet solution (Beyotime), and representative images are obtained with an optical microscope (Olympus, Japan). Finally, the counting of the relevant cells is completed by using Image J software.

### Establishment of ENH Stable Knockdown or Overexpression Cell Lines

We designed shRNA sequences specifically targeting ENH using the Thermo Fisher Scientific Design Website and cloned them into the cloning vector of shRNA‐pLKO.1‐puro. The ENH coding sequence was constructed into the lentiviral expression vector pLVX‐IRES‐puro. Subsequently, packaging plasmids pSPAX2, pMD2.G, and pLKO.1‐shENH or pLVX‐ENH were co‐transfected into 293T cells using Lipo3000 for lentivirus production. Lentiviruses were harvested 48 h after transfection and subsequently infected with LUAD cells. After 48 h of infection, lentiviruses were injected with 2 µg mL^−1^ puromycin incubation for 2 weeks to screen for stably infected LUAD cells. Western blotting was used to detect the efficiency of ENH knockdown or overexpression.

### Tumor Model

The 4–6 weeks‐old C57BL/6 mice were subcutaneously injected with ≈1   × 10^6^ control, ENH stable knockdown, or ENH stable overexpression LLC cells resuspended in 200 µL sterile PBS to develop tumors. Mice were treated with clodronate liposomes (Yeasen, Shanghai, China; i.p., 200 µL, every 3 days), cisplatin (Yeasen, Shanghai, China; i.v., 4 mg kg^−1^, every 3 days), maraviroc (MCE, Shanghai, China; i.p., 10 mg kg^−1^, every other day), or corresponding controls. Clodronate liposomes were injected 24 h before tumor cell implantation, while cisplatin and maraviroc were injected when the xenografts became palpable. Tumor volumes were measured every other day and calculated using the formula: Volume  =  L × W^2^/2 (L represents longest diameter; W represents shortest diameter). When tumors reached ≈2 cm, the mice were sacrificed, and tumors were harvested. All animal experiments were approved by the Animal Care and Use Committee of the Zhejiang University School of Medicine (Approval No. ZJU20220525). No samples, animals, or data were excluded from the experiment.

### Statistical Analysis

All experiments were conducted at least three times independently. Experimental data were statistically analyzed using Prism 8.0 software (GraphPad) and presented as mean ± S.D. Differences between two groups were assessed using Student's *t*‐test, and three or more groups were compared using one‐way analysis of variance (ANOVA) or two‐way ANOVA. A *p*‐value < 0.05 was considered statistically significant and assigned as p < 0.05 (*), p < 0.01 (**), or p < 0.001 (***).

Extended methodology including reagent validation and statistical approaches can be found in the Methods.

## Conflict of Interest

The authors declare no conflict of interest.

## Supporting information



Supporting Information

## Data Availability

The data that support the findings of this study are available from the corresponding author upon reasonable request.
