# Supplementary material for: Scaffolding Protein ENH Promotes Tumor Angiogenesis and Growth Through Macrophage Recruitment and Polarization
Source: Adv Sci (Weinh). 2025 Jun 19;12(34):e16476. doi: 10.1002/advs.202416476 (PMC12442595; doi:10.1002/advs.202416476)
Supplement: Supplementary file 1 — Supporting Information [file ADVS-12-e16476-s001.docx]

**Supplemental information**

**Scaffolding protein ENH promotes tumor angiogenesis and growth through macrophage recruitment and polarization**

Yueli Shi1,2#, Zhiyong Xu1,2#, Huan Wang3#, Bufu Tang4#, Nueraili Maihemuti1,2, Xinyuan Jiang1,2, Xiuying Chen5, Mingshu Xiao1,2, Sujing Jiang6, Yun Xu1,2, Peng Xiao7, Jiangnan Zhao1,2, Kaiyue Zhang1,2, Mengshu Li1,2, Kai Wang1,2*

1. Department of Respiratory and Critical Care Medicine, Center for Oncology Medicine, the Fourth Affiliated Hospital of School of Medicine, and International School of Medicine, International Institutes of Medicine, Zhejiang University, Yiwu, China, 322000

2. Zhejiang Key Laboratory of Precision Diagnosis and Treatment for Lung Cancer, Yiwu, China, 322000

3. Department of Respiratory & Critical Care Medicine, The First Affiliated Hospital of Nanjing Medical University, Nanjing, China

4. Department of Interventional Radiology, Zhongshan Hospital, Fudan University, Shanghai, China.

5. Department of Obstetrics and Gynecology, Center for Reproductive Medicine, the Fourth Affiliated Hospital of School of Medicine, and International School of Medicine, International Institutes of Medicine, Zhejiang University, Yiwu, China, 322000.

6. Department of Gastroenterology, The Second Affiliated Hospital of Wenzhou Medical University, Wenzhou, China, 325000

7. Sir Run Run Shaw Hospital, Zhejiang University School of Medicine, Hangzhou, China

*Corresponding author:

Kai Wang, Email: [kaiw@zju.edu.cn](mailto:kaiw@zju.edu.cn).

#These authors have contributed equally to this work.

**Supplementary Materials**

**Cell culture**

Human LUAD cell lines (A549, H1975, and H1650), mouse lung cancer cell line (LLC), and HEK293T cells were obtained from the American Type Culture Collection (Manassas, VA, USA). Our laboratory previously isolated human umbilical vein endothelial cells (HUVECs). A549, H1975, and H1650 cells were cultured in RPMI-1640 medium, while LLC and HEK293T cells were cultured in Dulbecco's Modified Eagle Medium. All media were supplemented with 10% fetal bovine serum and 1% penicillin-streptomycin-glutamine. HUVECs (passages 4–6) were cultured in M199 medium containing 37% human endothelial serum-free mixed medium, 10% FBS, and 15 μg/mL endothelial cell growth supplement. Cells were incubated in a 37°C humidified atmosphere with 5% CO_2_.

**Enzyme-linked immunosorbent assay (ELISA)**

The levels of secreted CCL5 in cell culture supernatants and human serum samples were measured using ELISA kits from ABclonal (Wuhan, China) according to the manufacturer’s instructions.

**Quantitative polymerase chain reaction (qPCR)**

Total RNA was extracted using the RNA-Quick Purification Kit (YiShan, Shanghai, China) according to the manufacturer's instructions. 1 μg of total RNA was reverse-transcribed to cDNA using the ReverTra Ace™ qPCR RT kit (Toyobo, Osaka, Japan). Gene expression was analyzed by real-time PCR using SYBR green dye with CFX96 Touch Real-Time PCR Detection System (Bio-Rad). Gene expression levels were calculated using the equation RQ = 2^−△△Ct^, normalized to 18S RNA or GAPDH. Primers used for qPCR are listed in Supplementary Table S1 and 2.

**Tube formation assay**

The u-Slide wells (Ibidi, Martinsried, Germany) coated with 10 μL of Matrigel were incubated at 37°C until solidified. After solidification, 1 × 10^4^ HUVECs were resuspended in the indicated conditioned media (CM) from LUAD cells and seeded into the u-Slide. The u-Slide was incubated at 37°C for 4h, then visualized and captured using an inverted microscope. Total tube length was analyzed using ImageJ software by determining the average of five random fields per group.

**Co-immunoprecipitation (CO-IP) and immunoblotting**

Total proteins were extracted on ice using RIPA lysis buffer (Beyotime, Shanghai, China) containing protease and phosphatase inhibitors. Proteins were separated by SDS-PAGE and transferred to nitrocellulose membranes. Membranes were blocked with 5% skimmed milk and incubated overnight at 4°C with the indicated primary antibody. After probed with horseradish peroxidase-conjugated secondary antibody, the membranes were visualized using a chemiluminescence substrate kit. Antibody information is provided in Supplementary Table 3.

For CO-IP, cells were lysed in Nonidet P-40 buffer (Beyotime, Shanghai, China), and the lysates were incubated overnight at 4°C with antibody-conjugated magnetic beads. Immunoprecipitates were eluted at 100°C for 5 min and subjected to immunoblotting.

**RNA-sequence analysis**

Total RNA was extracted from ENH knockdown and corresponding control A549 cells. Library construction, sequencing, and transcriptome analysis were performed by Biomarker Technology (Beijing, China).

**Immunofluorescence (IF) and immunohistochemistry (IHC) Staining**

Paraffin-embedded tissue samples were cut into 4 μm thick sections. The sections were deparaffinized and subjected to antigen retrieval according to the recommendations of Cell Signaling Technology (Danvers, MA, USA). Sections were incubated overnight at 4°C with the corresponding primary antibody (Supplementary Table 1). Alexa Fluor 555, 546 or 488 fluorescent secondary antibodies were used for IF, and peroxidase-conjugated secondary antibodies for IHC to detect the binding of primary antibodies. IF images were captured using a confocal microscope (FV3000, Olympus), and IHC images using a light microscope. Fluorescence intensity and IHC scores were determined using Image J software. Counts of infiltrated TAMs were determined by the number of F4/80^+^ or CD68^+^ cells per field using Image J software. Microvessel density (MVD) was calculated based on CD31 staining. All calculations were performed by determining the average of five random fields.

**Flow cytometry**

Tumors were cut up and digested with collagenase IV in a shaking table at 37°C for 1 h. Cell suspensions were filtered at 4°C and 400×g for 10 min, then resuspended in PBS. Cells in suspension were blocked with anti-CD16/CD32 and labeled with a dead cell staining kit (BioLegend, CA, USA). Cells were then incubated with corresponding fluorochrome-labeled antibodies (Biolegend, CA, USA) for 30 min at 4°C in the dark. Flow cytometry was performed using a CytoFLEX Flow Cytometer (Beckman Coulter).

**Bioinformatics analysis**

Expression correlations between ENH, PECAM1, CCL5, and macrophage and monocyte markers were analyzed using the Gene Expression Profiling Interactive Analysis online database (<http://gepia2.cancer-pku.cn>). The Tumor Immune Estimation Resource (TIMER) database (<http://timer.cistrome.org/>) was used to explore the relationship between the gene signature and immune cell infiltration. mRNA-seq data and scRNA-seq data were obtained from Gene Expression Omnibus (GEO, <https://www.ncbi.nlm.nih.gov/geo/index.cgi>) and The Cancer Genome Atlas (TCGA, <https://cancergenome.nih.gov/>) databases. Survival estimates in patients with LUAD were assessed using the Kaplan–Meier plotter and PrognoScan ([https://kmplot.com](https://kmplot.com/) and <http://gibk21.bse.kyutech.ac.jp/PrognoScan/index.html>, respectively) databases. ENH interaction networks were analyzed using the BioGRID (<https://thebiogrid.org/>) database. VEGFA expression in each cell population in NSCLC was analyzed using the TISCH (http://[[TISCH (comp-genomics.org)](http://tisch1.comp-genomics.org/))](http://tisch.comp-genomics.org/home/) database.

**Supplemental tables**

**Table S1. The primers used for qPCR analysis (human)**

| **Primer Name** | **Primer Sequence 5’-3’** |
| --- | --- |
| ENH-F | TCCTTGGAGAAGTCATCAATGC |
| ENH-R | CACCATCCTCCAAGTGAAAAAC |
| PECAM1-F | AACAGTGTTGACATGAAGAGCC |
| PECAM1-R | TGTAAAACAGCACGTCATCCTT |
| VEGFA-F | GATGAGCTTCCTACAGCACAACAA |
| VEGFA-R | TTTCGTTTTTGCCCCTTTCC |
| MMP9-F | CATTTCGACGATGACGAGTTGT |
| MMP9-R | CGGGTGTAGAGTCTCTCGC |
| FGF2-F | AGAAGAGCGACCCTCACATCA |
| FGF2-R | CGGTTAGCACACACTCCTTTG |
| PDGF-BB-F | CTCGATCCGCTCCTTTGATGA |
| PDGF-BB-R | CGTTGGTGCGGTCTATGAG |
| ANGPT1-F | AGCGCCGAAGTCCAGAAAAC |
| ANGPT1-R | TACTCTCACGACAGTTGCCAT |
| IL8-F | ACTGAGAGTGATTGAGAGTGGAC |
| IL8-R | AACCCTCTGCACCCAGTTTTC |
| VWF-F | CCGATGCAGCCTTTTCGGA |
| VWF-R | TCCCCAAGATACACGGAGAGG |
| ANGPT2-F | AACTTTCGGAAGAGCATGGAC |
| ANGPT2-R | CGAGTCATCGTATTCGAGCGG |
| MMP7-F | GAGTGAGCTACAGTGGGAACA |
| MMP7-R | CTATGACGCGGGAGTTTAACAT |
| CCL5-F | CCAGCAGTCGTCTTTGTCAC |
| CCL5-R | CTCTGGGTTGGCACACACTT |
| IL10-F | TCAAGGCGCATGTGAACTCC |
| IL10-R | GATGTCAAACTCACTCATGGCT |
| CD163-F | TTTGTCAACTTGAGTCCCTTCAC |
| CD163-R | TCCCGCTACACTTGTTTTCAC |
| YAP-F | TAGCCCTGCGTAGCCAGTTA |
| YAP-R | TCATGCTTAGTCCACTGTCTGT |
| GAPDH-F | CAGGTGGTCTCCTCTGACTTCAA |
| GAPDH-R | ACCCTGTTGCTGTAGCCAAATTC |
| 18S-F | GTAACCCGTTGAACCCCATT |
| 18S-R | CCATCCAATCGGTAGTAGCG |

**Table S2. The primers used for qPCR analysis (mouse)**

| **Primer Name** | **Primer Sequence 5’-3’** |
| --- | --- |
| VEGFA-F | GGAGACTCTTCGAGGAGCACTT |
| VEGFA-R | GGCGATTTAGCAGCAGATATAAGAA |
| MMP9-F | CTGGACAGCCAGACACTAAAG |
| MMP9-R | CTCGCGGCAAGTCTTCAGAG |
| FGF2-F | GAGTTGTGTCTATCAAGGGAGTG |
| FGF2-R | CCGTCCATCTTCCTTCATAGC |
| PDGF BB-F | CATCCGCTCCTTTGATGATCTT |
| PDGF BB-R | GTGCTCGGGTCATGTTCAAGT |
| ANGPT1-F | CACATAGGGTGCAGCAACCA |
| ANGPT1-R | CGTCGTGTTCTGGAAGAATGA |
| CXCL15-F | CAAGGCTGGTCCATGCTCC |
| CXCL15-R | TGCTATCACTTCCTTTCTGTTGC |
| VWF-F | CTTCTGTACGCCTCAGCTATG |
| VWF-R | GCCGTTGTAATTCCCACACAAG |
| ANGPT2-F | CCTCGACTACGACGACTCAGT |
| ANGPT2-R | TCTGCACCACATTCTGTTGGA |
| MMP7-F | CTGCCACTGTCCCAGGAAG |
| MMP7-R | GGGAGAGTTTTCCAGTCATGG |
| CCL5-F | GCTGCTTTGCCTACCTCTCC |
| CCL5-R | TCGAGTGACAAACACGACTGC |
| ENH-F | TGTGTCATTGGTCGGCCC |
| ENH-R | CTTGCCACCATCCTTCAGACT |
| GAPDH-F | CTTCACCACCATGGAGAAGGC |
| GAPDH-R | GGCATGGACTGTGGTCATGAG |

**Table S3. Antibody**

| **Antibody** | **Cat number** | **Dilution** | **Source** | **Company** |
| --- | --- | --- | --- | --- |
| Anti-PDLIM5 | ab196559 | WB: 1:1000 IF/IHC/IP: 1:100 | Rabbit | Abcam |
| Anti-Flag | AF519 | WB: 1:1000  IP: 1:100 | Mouse | Beyotime |
| Anti-Flag | #14793 | WB: 1:1000  IP: 1:100 | Rabbit | Cell Signaling |
| Anti-Myc | TA150121 | WB: 1:1000  IP: 1:100 | Mouse | ORIGENE |
| Anti-Myc | #2278 | WB: 1:1000 | Rabbit | Cell Signaling |
| Anti-HA Tag | E022010 | WB: 1:1000  IP: 1:100 | Mouse | EarthOx |
| Anti-HA | db5297 | WB: 1:1000 | Rabbit | Diagbio |
| Anti-PCNA | #13110 | WB: 1:1000 | Rabbit | Cell Signaling |
| Anti-Cyclin D1 | #2922 | WB: 1:1000 | Rabbit | Cell Signaling |
| Anti-CD31 | AF3628 | IF: 1:200 | Goat | R&D system |
| Anti-CD31 | ab28364 | IF: 1:200 | Rabbit | Abcam |
| Anti-CD68 | [TD7518](http://www.ab-mart.com.cn/page.aspx?node=%2077%20&id=%2023363) | IHC: 1:100 | Rabbit | Abmart |
| Anti-F4/80 | #70076 | IHC: 1:150 | Rabbit | Cell Signaling |
| Anti-CCL5 | #36467 | IHC: 1:50 | Rabbit | Cell Signaling |
| Anti-p-STAT3 | ab76315 | WB: 1:1000 | Rabbit | Abcam |
| Anti-STAT3 | ab109085 | WB: 1:1000 | Rabbit | Abcam |
| Anti-YAP | #14074 | WB: 1:1000  IF: 1:100 | Rabbit | Cell Signaling |
| Anti-p-YAP | #13008 | WB: 1:1000 | Rabbit | Cell Signaling |
| Anti-KPNA2 | T57210 | WB: 1:1000 | Rabbit | Abmart |
| Anti-β Tubulin | db3285 | WB: 1:3000 | Rabbit | Diagbio |
| Anti-Lamin B1 | [db6419](http://www.diagbio.com/product/PNOdb6419.html) | WB: 1:1000 | Mouse | Diagbio |
| Anti-β-Actin | M1210-2 | WB: 1:5000 | Mouse | HuaBio |
| 800CW Goat anti-Rabbit (WB) | 925-32211 | 1:5000 | Goat | LI-COR |
| 680RD Goat anti-Mouse (WB) | 925-68070 | 1:5000 | Goat | LI-COR |
| Goat anti-Rabbit, Alexa Fluor 546 (IF) | A-11035 | 1:200 | Goat | ThermoFisher |
| Goat anti-Rabbit, Alexa Fluor 488 (IF) | A-11008 | 1:200 | Goat | ThermoFisher |
| Donkey anti-Goat, Alexa Fluor 555 (IF) | A-21432 | 1:200 | Donkey | ThermoFisher |

**Supplementary Figures**

**
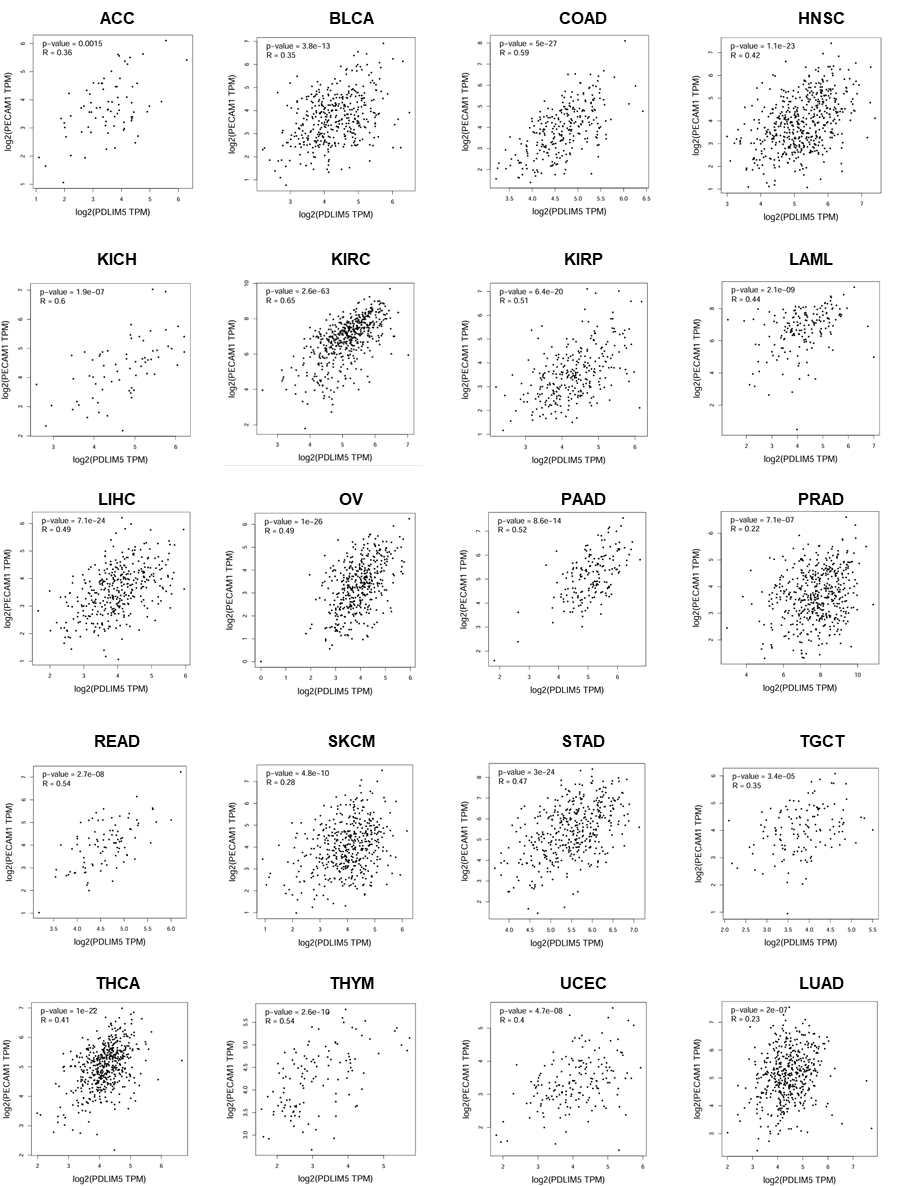
**

**Figure S1. ENH is positively correlated with MVD in a range of tumor tissues**

Correlation between ENH and PECMA1 in pan-cancer was analyzed using the GEPIA database.

**
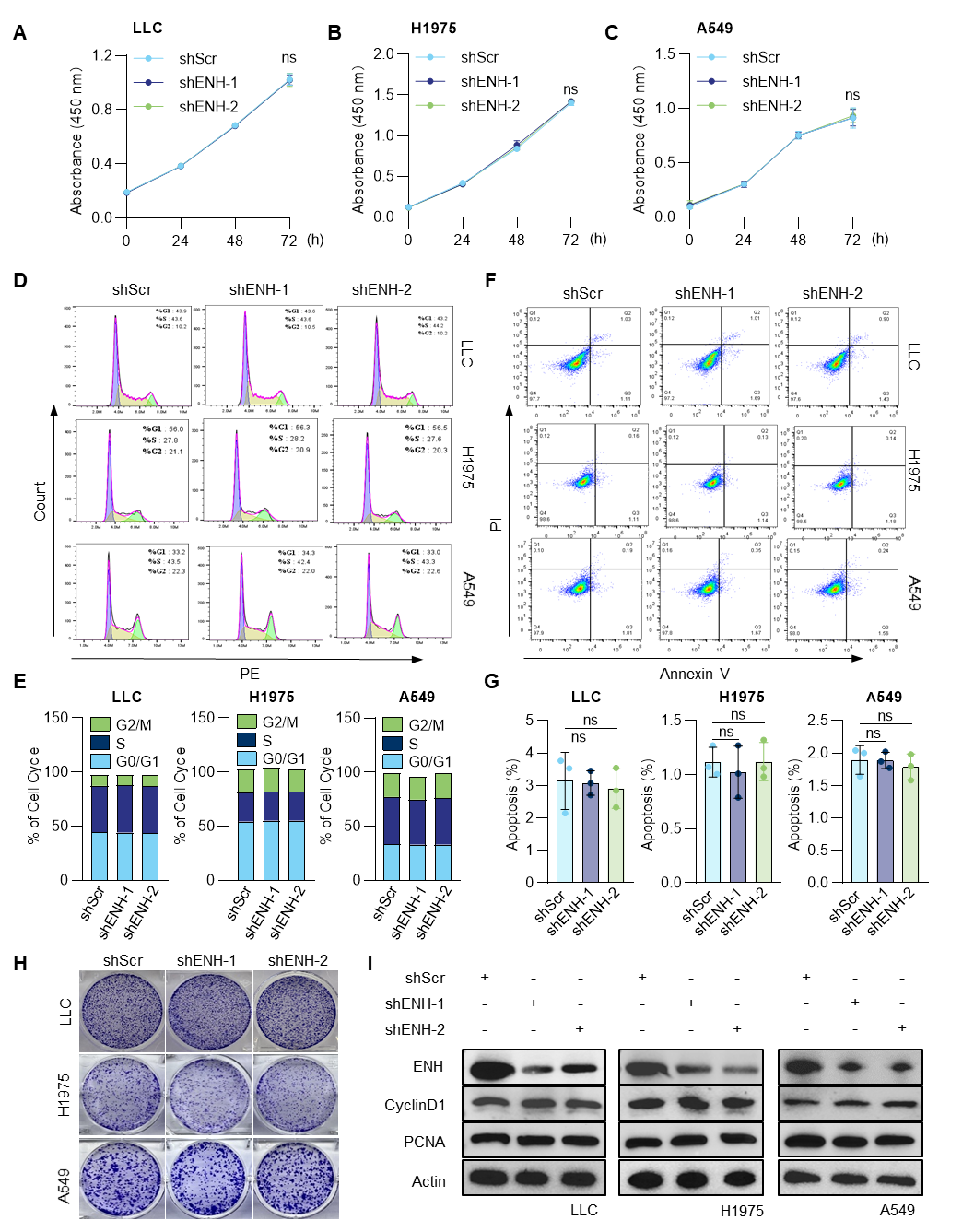
**

**Figure S2. Cell proliferation is unaffected with ENH disruption**

A-C. Proliferation ability of lung cancer cells with ENH knockdown was detected by CCK8 (n=6).

D, E. Cell cycle was analyzed by flow cytometry on ENH knockdown lung cancer cells, and the percentage of cell cycle phases was shown as a bar graph (n=3).

F, G. Cell apoptosis was detected by flow cytometry on ENH knockdown lung cancer cells, and the quantification of cell apoptosis rate was shown as a bar graph (n=3).

1. Clonogenic assay of lung cancer cells with ENH knockdown.
2. Western blot analysis of cell proliferation related proteins in ENH knockdown lung cancer cells.


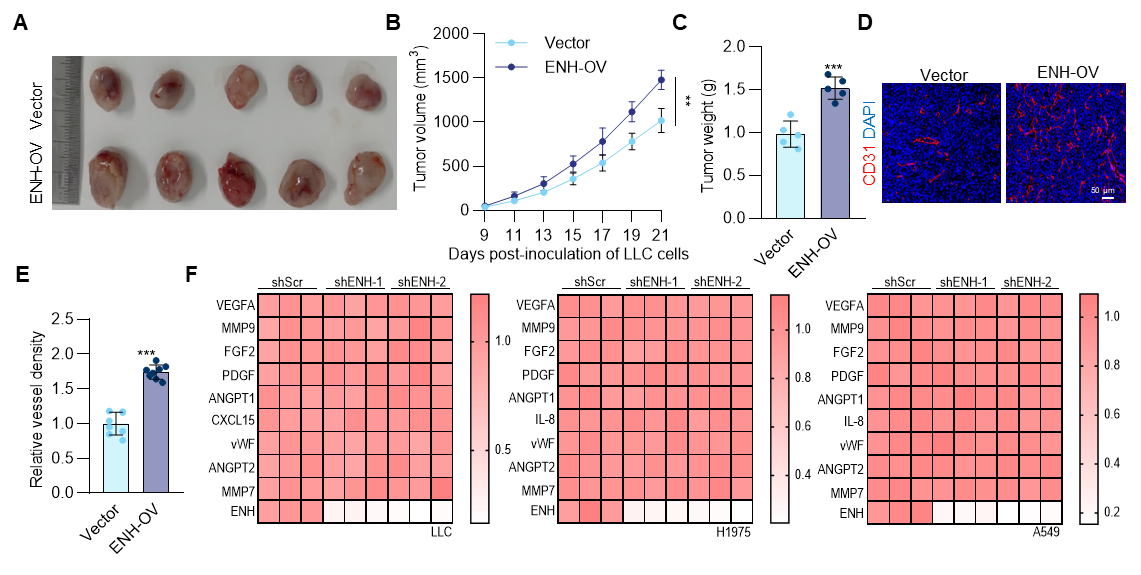


**Figure S3. ENH regulates tumor angiogenesis not by altering the expression of known pro-angiogenic genes**

1. Tumor tissue from each group collected 21 days after injection of LLC cells (n=5).
2. Subcutaneous tumor growth of LLC cells stably transduced with plasmid vector (Vector) or plasmid with ENH overexpression (ENH-OV) (n=5).
3. Tumor weight of LLC murine model in ENH overexpression or control group at day 21 after tumor injection (n=5).
4. Representative images of IF staining for CD31 in ENH overexpression LLC subcutaneous tumors sections were shown.
5. Quantification of CD31^+^ vessels density was shown (n=8).
6. The heat map of relative mRNA expression level of well-known angiogenic genes in lung cancer cells with ENH knockdown.


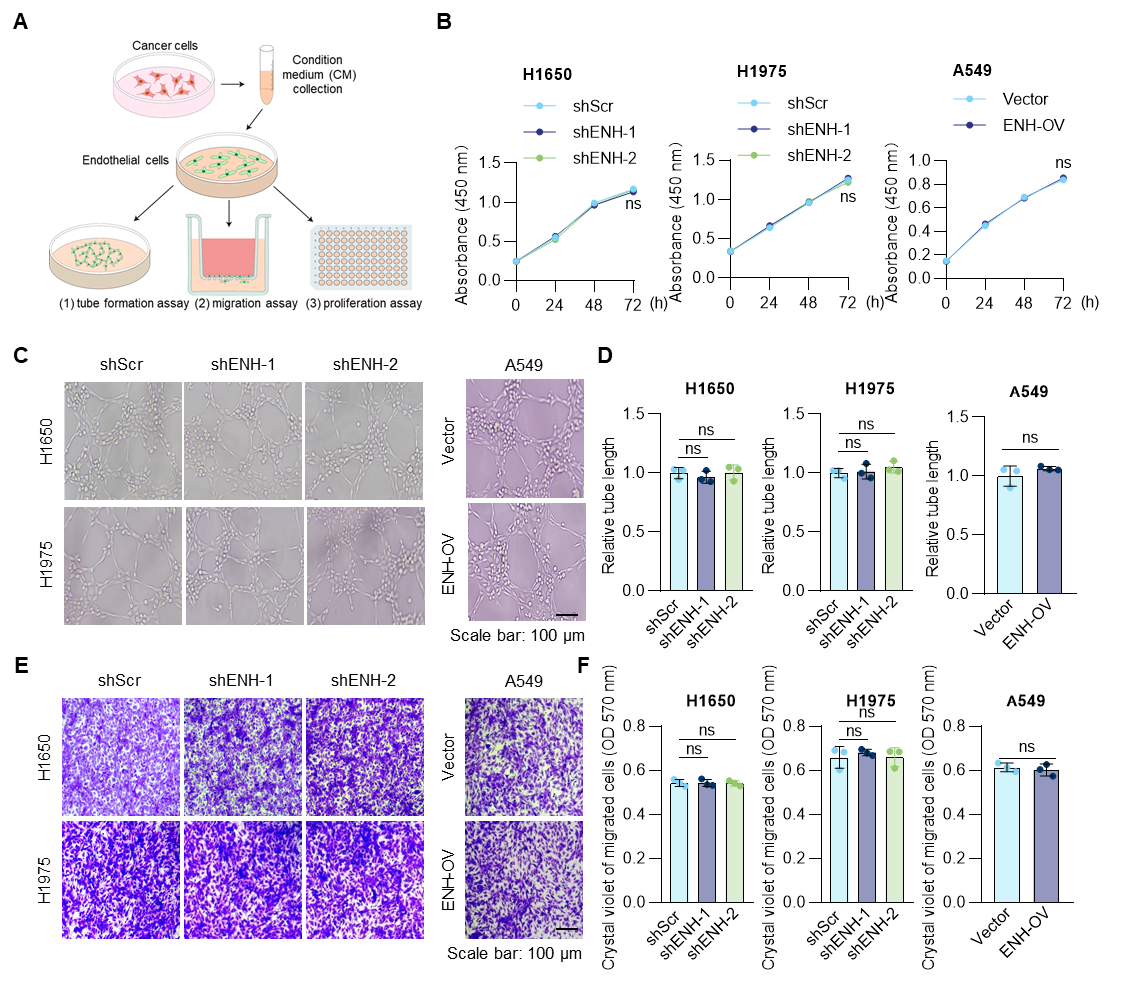


**Figure S4. Supernatants from ENH knockdown or overexpression cells have no significant effect on the angiogenic capacity of endothelial cells**

1. Schematic overview of functional assays of HUVECs after treatment with CM isolated from LUAD cells.

B-F. HUVECs treated with CM collected from ENH knockdown or overexpression LUAD cells，then (B) CCK8 assay was used to detected HUVECs proliferation ability (n=6); (C) Tube formation assay was used to detected HUVECs tube formation ability, tube lengths (D) were measured by using the Image J software and shown as mean±SD (n=3); (E) Transwell assay was used to detected HUVECs migration ability, and migration index (F) was quantified (n=3).


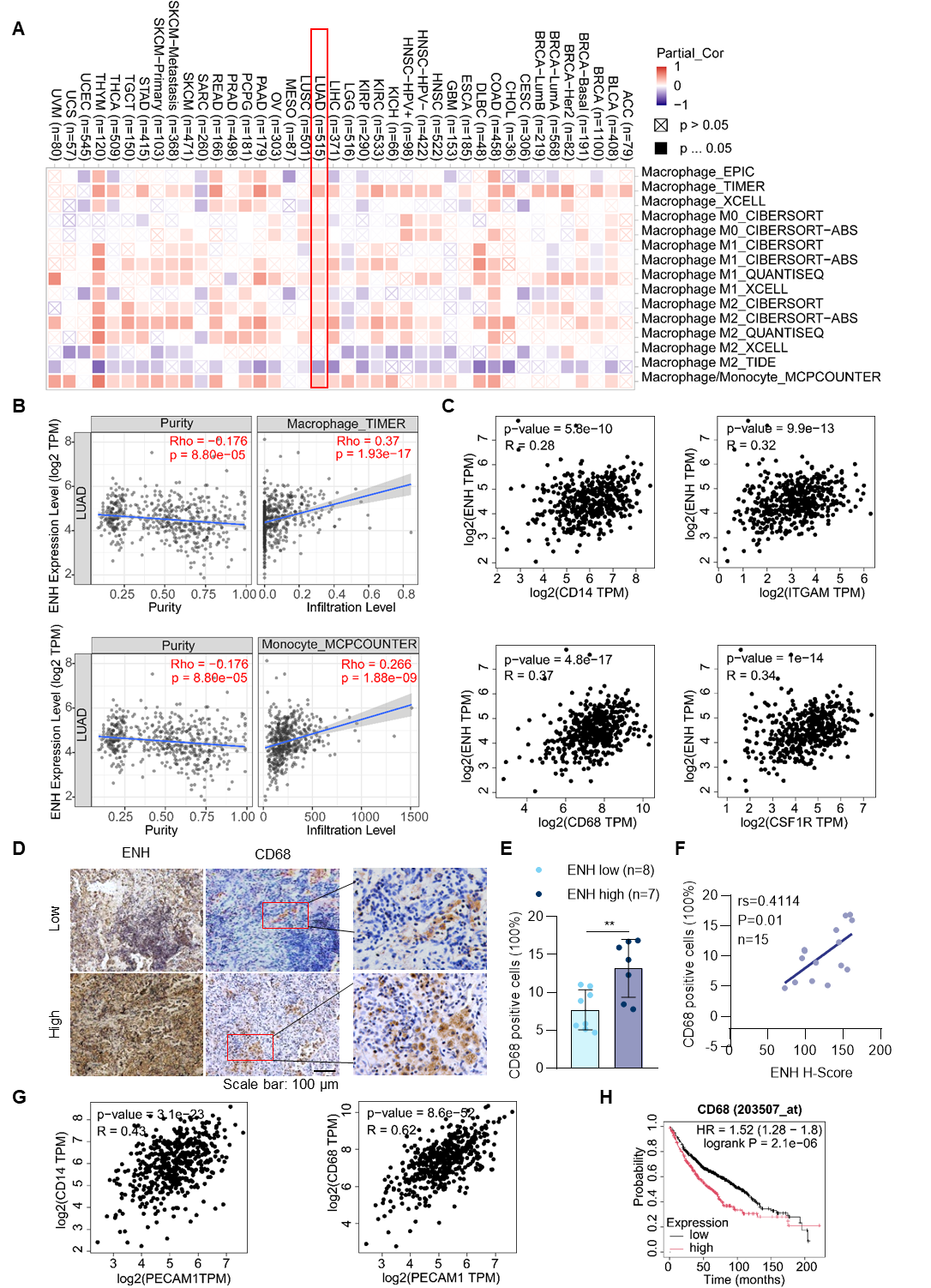


**Figure S5. The content of TAMs is significantly correlated with ENH levels and MVD**

1. Correlation of ENH with TAMs infiltration in all tumor types was analyzed using the TIMER 2.0 database.
2. Correlation of ENH with TAMs and monocytes infiltration in LUAD was analyzed using the TIMER 2.0 database.
3. Correlation of ENH with macrophage and monocyte markers in LUAD was analyzed using the GEPIA database.
4. Representative images of IHC staining for ENH and CD68 in human LUAD tissues were shown.
5. Percentages of CD68^+^ cells in LUAD were analyzed using Image J software, and the results were presented in two groups according to low and high ENH expression (ENH low:8, ENH high:7).
6. Identification of the correlation between ENH and percentages of CD68^+^ cells in 15 LUAD tissues based on the IHC results.
7. Correlation of PECAM1 with macrophage and monocyte markers in LUAD was analyzed using the GEPIA database.
8. Kaplan–Meier analysis of OS of LUAD patients based on CD68 expression.


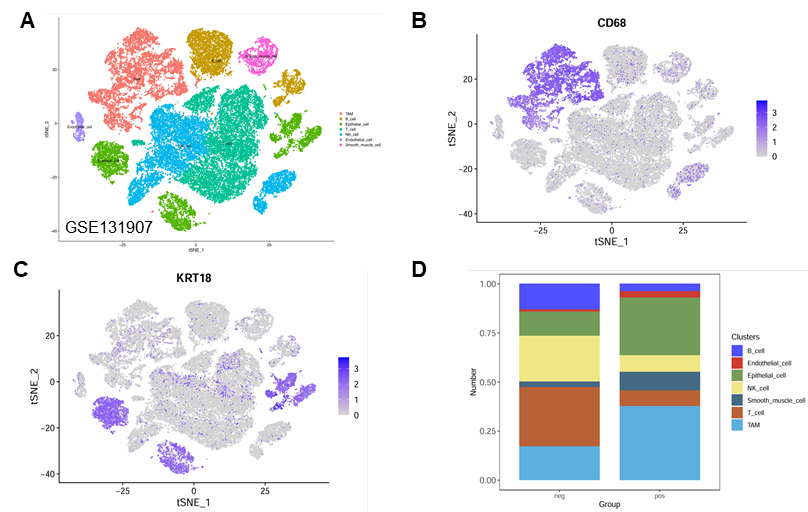


**Figure S6. Cell proportions relevant to ENH level**

A. The cellular landscape of LUAD in the GSE131907.

B, C. Expression profiles of CD68 and KRT18.

D. Mean proportions of the 7 cell types in PDLIM5 negative and positive groups.
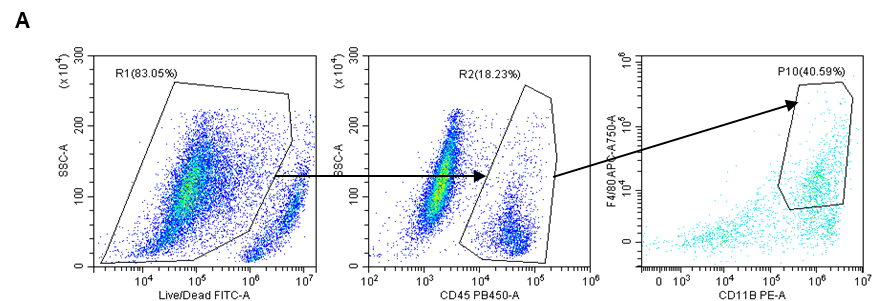


**Figure S7. Gating strategy to identify TAMs**

1. The gating strategy to identify CD45^+^CD11b^+^F4/80^+^ macrophages in mouse tumors.


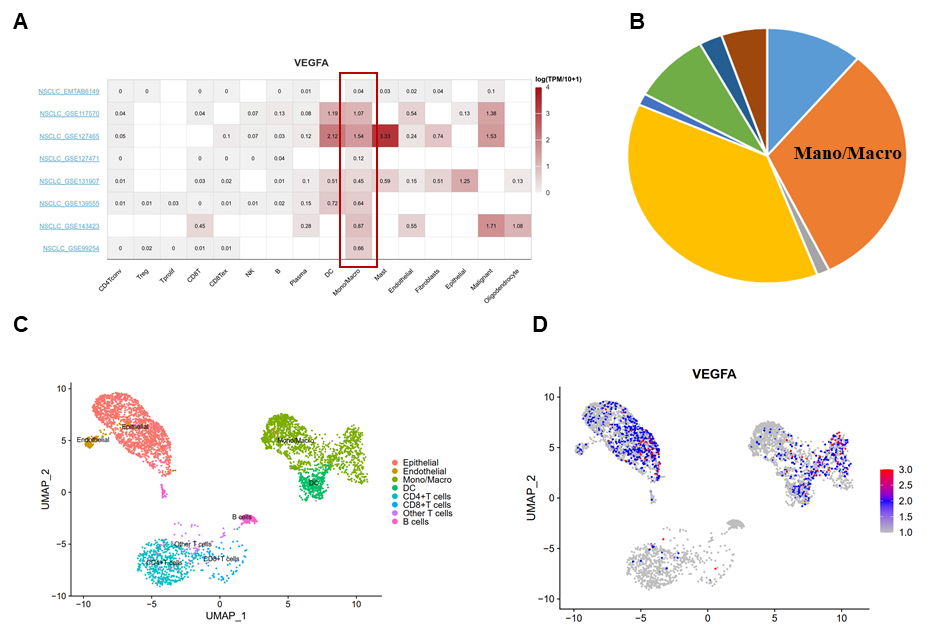


**Figure S8. VEGFA is mainly expressed in TAMs.**

1. Heatmap of VEGFA expression in different cell types in NSCLC from the TISCH database.
2. The cell types and their percentage in the GSE117570 dataset.

C, D. The distribution of VEGFA in different cell types in the GSE117570 dataset.


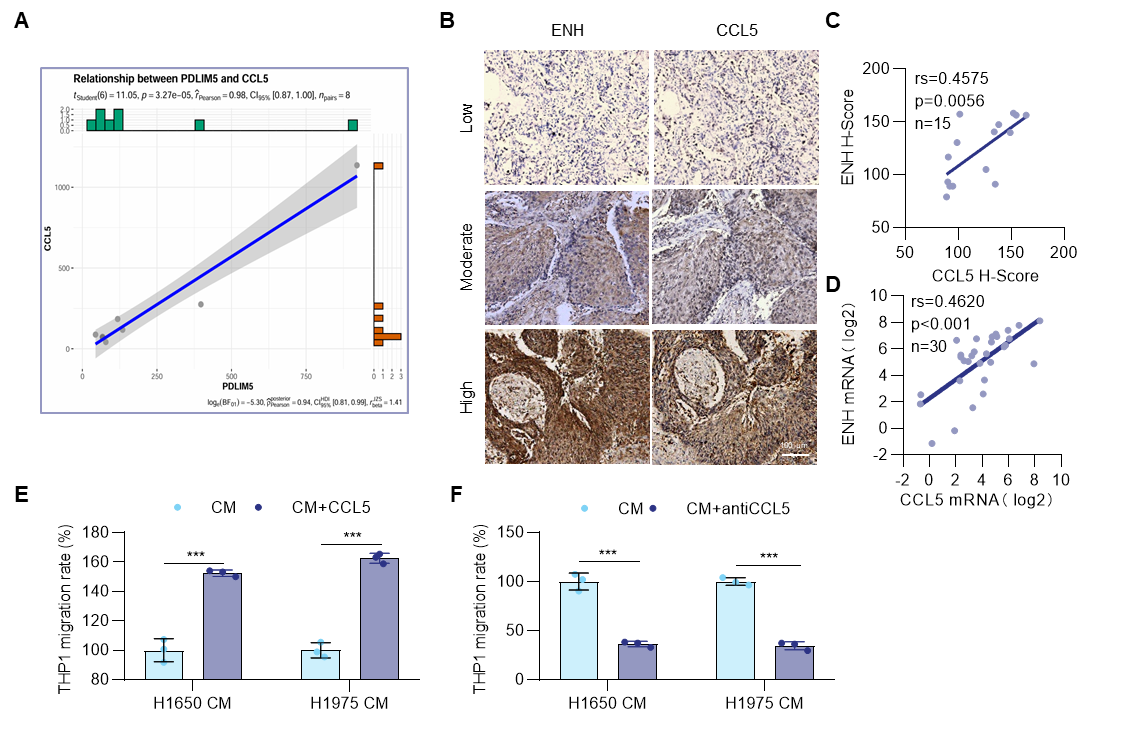


**Figure S9. ENH was positively correlated with CCL5 expression**

1. Correlation between ENH and CCL5 in LUAD was analyzed using the GEO datasets (GSE85841).
2. Representative images of IHC staining for ENH and CCL5 in human LUAD tissues were shown.
3. Identification of the correlation between ENH and CCL5 in 15 LUAD tissues based on the IHC results (n=15).
4. Identification of the correlation between ENH and CCL5 mRNAs in 30 LUAD tissues by qPCR.
5. CM collected from H1650 and H1975 cells added with or without rCCL5 protein was used as chemoattractants in THP1 chemotaxis assay. The bar graph showed the number of migrated THP1 cells, represented as relative percentage to the control (n=3).
6. CM collected from H1650 and H1975 cells added with or without antiCCL5 neutralizing antibody was used as chemoattractants in THP1 chemotaxis assay. The bar graph showed the number of migrated THP1 cells, represented as relative percentage to the control (n=3).


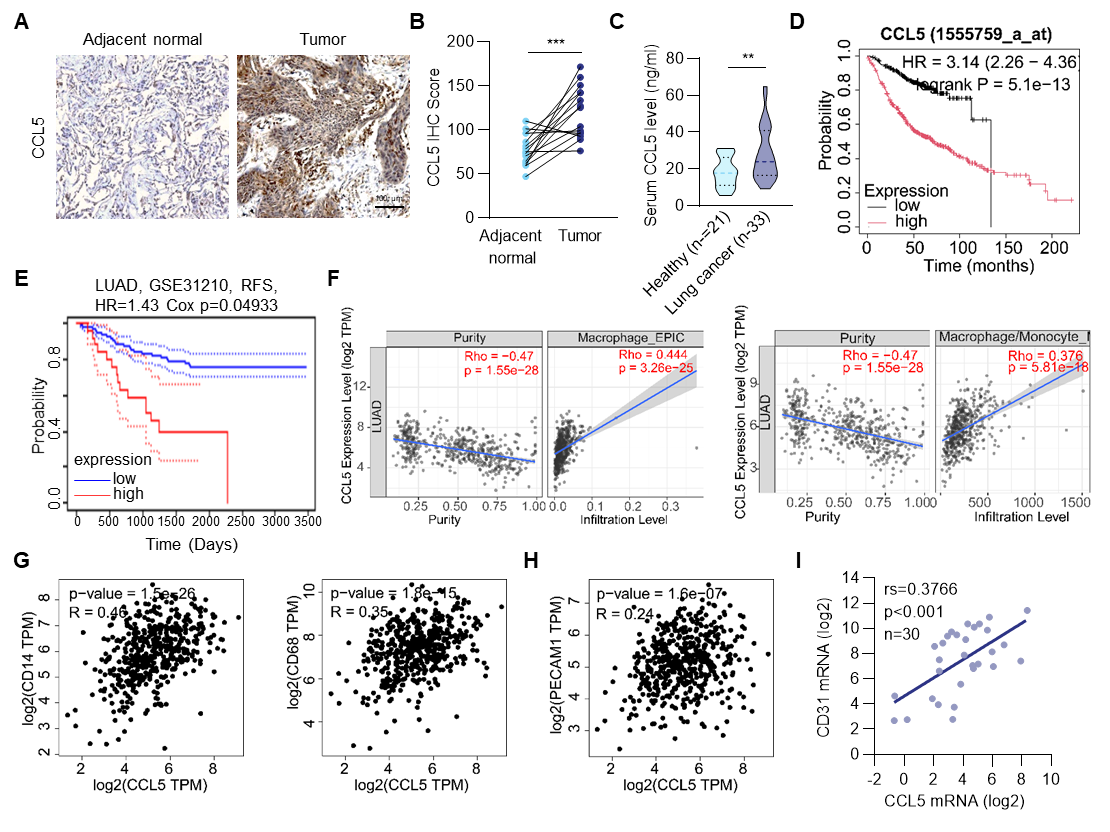


**Figure S10. CCL5 upregulated in LUAD patients is correlated with TAMs levels and MVD**

1. Representative images of IHC staining for CCL5 in human LUAD tissues and adjacent normal tissues were shown.
2. Statistical analysis of IHC results of CCL5 expression in human LUAD tissues and adjacent normal tissues was shown as a bar graph (n=15).
3. The levels of CCL5 in serum from LUAD patients or normal populations were measured by ELISA (LUAD:33, Healthy:21).
4. Kaplan–Meier analysis of OS of LUAD patients based on CCL5 expression.
5. Kaplan–Meier analysis of RFS of LUAD patients based on CCL5 expression.
6. Correlation of CCL5 with TAMs and monocytes infiltration in LUAD was analyzed using the TIMER 2.0 database.
7. Correlation of CCL5 with macrophage and monocyte markers in LUAD was analyzed using the GEPIA database.
8. Correlation of CCL5 with PECAM1 in LUAD was analyzed using the GEPIA database.
9. Identification of the correlation between CD31 and CCL5 mRNAs in 30 LUAD tissues by qPCR.


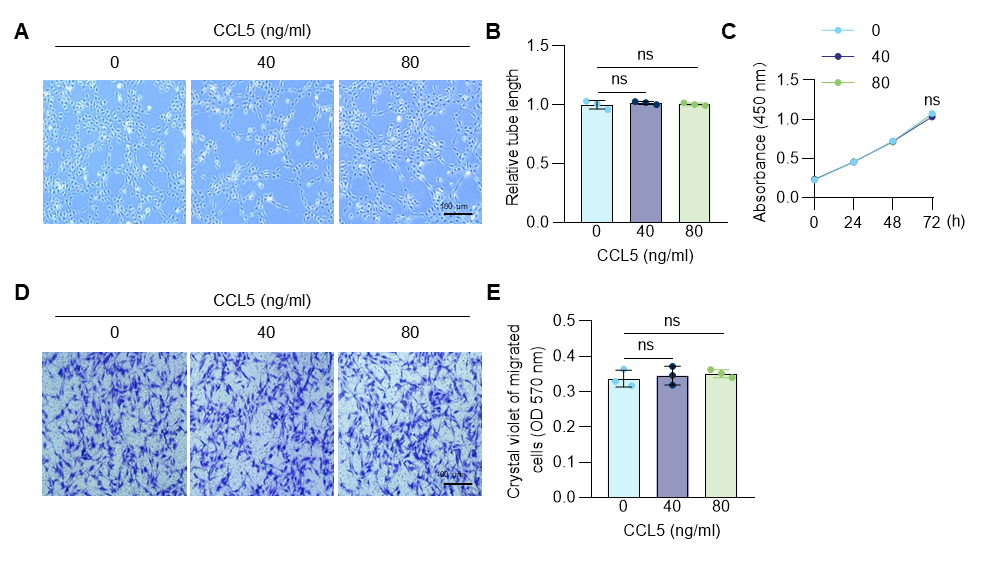


**Figure S11. CCL5 fails to directly enhance endothelial angiogenesis**

A-E. HUVECs treated with or without CCL5 protein, then (A) Tube formation assay was used to detected HUVECs tube formation ability, tube lengths (B) were measured by using the Image J software and shown as mean ±SD (n=3); (C) CCK8 assay was used to detected HUVECs proliferation ability (n=6); (D) Transwell assay was used to detected HUVECs migration ability, and migration index (E) was quantified (n=3).


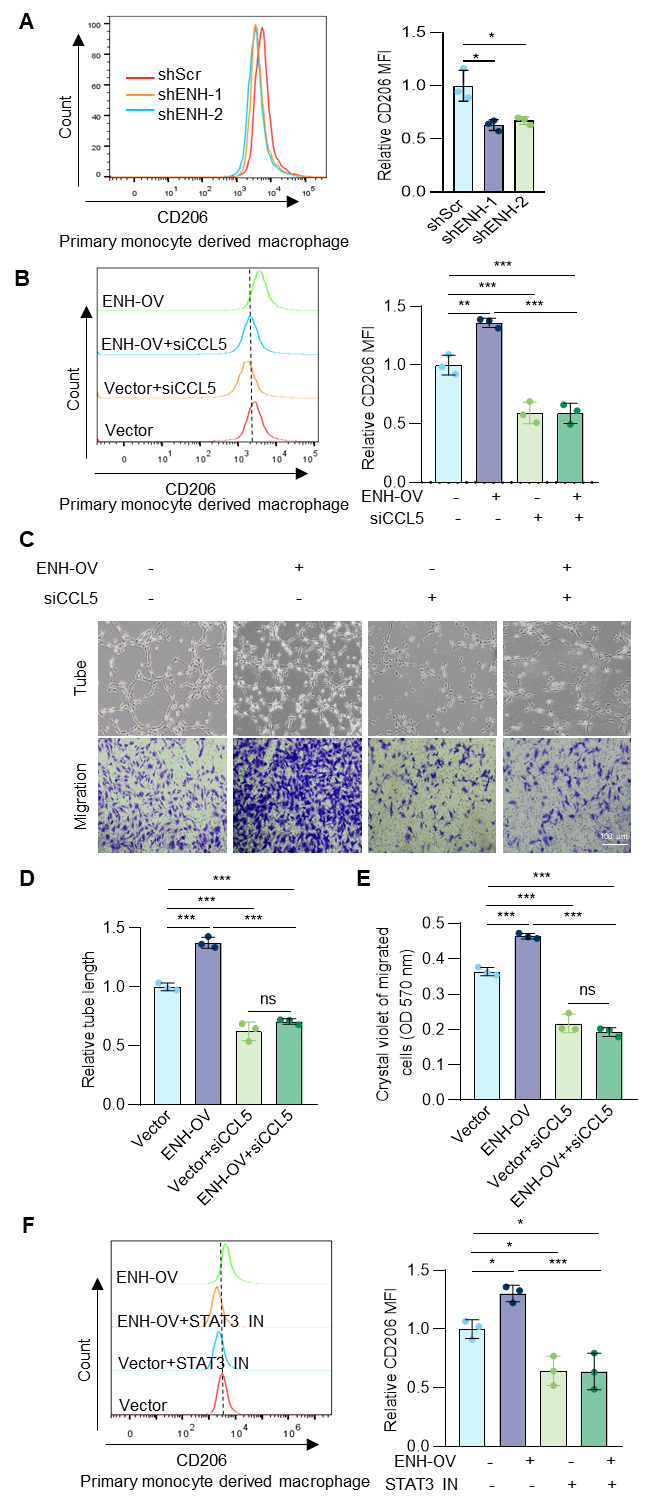


**Figure S12. ENH-induced M2-polarized macrophages are capable of promoting angiogenesis**

1. Flow cytometric analysis of CD206 expression in human primary monocytes-derived macrophages co-cultured with ENH knockdown LUAD cells. The mean fluorescence intensities (MFI) value of each group was represented in histogram (n=3).
2. Flow cytometric analysis of CD206 expression in human primary monocytes-derived macrophages co-cultured with ENH overexpression and CCL5 knockdown LUAD cells. The MFI value of each group was represented in histogram (n=3).

C-E. The supernatants collected from THP1-drived macrophages co-cultured with ENH overexpression and CCL5 knockdown LUAD cells were used as CM. HUVECs treated with indicated CM, then (C) Tube formation assay was used to detected HUVECs tube formation ability, tube lengths (D) were measured by using the Image J software and shown as mean ±SD (n=3); (C) Transwell assay was used to detected HUVECs migration ability, and migration index (E) was quantified (n=3).

F. Flow cytometric analysis of CD206 expression in human primary monocytes-drived macrophages co-cultured with ENH overexpression LUAD cells treated with or without STAT3 inhibitor (STAT3-IN). The MFI value of each group was represented in histogram (n=3).


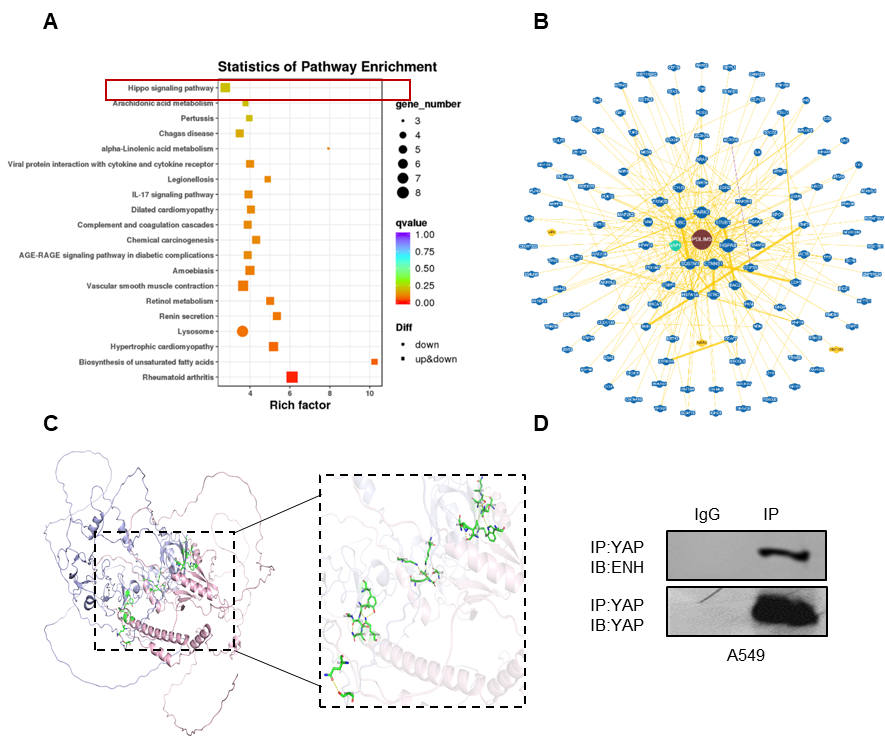


**Figure S13. ENH involved in Hippo-YAP signaling pathway.**

A. The KEGG pathway enrichment analysis of DEGs derived from RNA-seq data.

B. ENH interaction network analyzed using the BioGRID online database.

C. Molecular docking pattern diagram of ENH and YAP.

D. CO-IP analysis of the endogenous interaction between ENH and YAP in A549 cells.

**
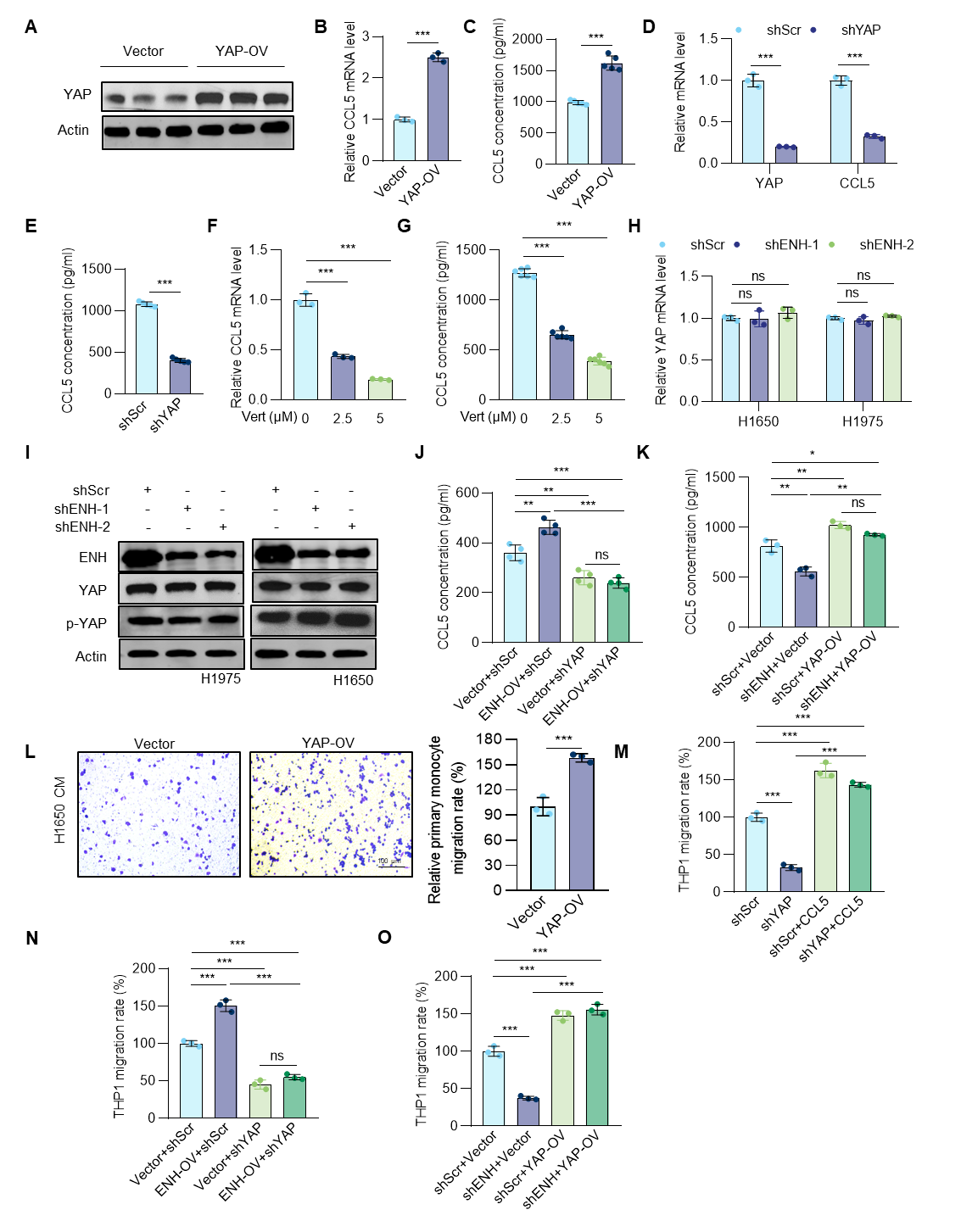
Figure S14. YAP regulates CCL5 expression and macrophage recruitment**

1. Efficiency of YAP overexpression in H1650 cells was detected by western blot analysis.
2. qPCR analysis of CCL5 mRNA levels in YAP overexpression H1650 cells (n=3).
3. ELISA analysis of CCL5 protein levels in YAP overexpression H1650 cells (n=5).
4. qPCR analysis of CCL5 mRNA levels in YAP knockdown H1650 cells (n=3).
5. ELISA analysis of CCL5 protein levels in YAP knockdown H1650 cells (n=5)*.*
6. qPCR analysis of CCL5 mRNA levels in H1650 cells treated with indicated concentration of YAP inhibitor (Verteporfin, Vert) for 24 h (n=3).
7. ELISA analysis of CCL5 protein levels in H1650 cells treated with indicated Verteporfin for 24 h (n=6).
8. qPCR analysis of YAP mRNA levels in ENH knockdown H1650 and H1975 cells (n=3).
9. Western blot analysis of YAP/p-YAP levels in ENH knockdown H1650 and H1975 cells.
10. ELISA analysis of CCL5 protein levels in ENH overexpression A549 cells with or without *YAP* knockdown (n=4).
11. ELISA analysis of CCL5 protein levels in ENH knockdown H1650 cells with or without *YAP* overexpression (n=3).
12. Representative images and a bar graph depicted the quantity of migrated human primary monocytes stimulated by CM isolated from YAP overexpression H1650 cells. The results were presented as a relative percentage compared to the control group (n=3).
13. CM collected from YAP knockdown H1650 cells added with or without rCCL5 protein was used as chemoattractants in THP1 chemotaxis assay. The bar graph showed the number of migrated THP1 cells, represented as relative percentage to the control (n=3).
14. CM collected from ENH overexpression A549 cells with or without YAP knockdown was used as chemoattractants in THP1 chemotaxis assay. The bar graph showed the number of migrated THP1 cells, represented as relative percentage to the control (n=3).
15. CM collected from ENH knockdown H1650 cells with or without YAP overexpression was used as chemoattractants in THP1 chemotaxis assay. The bar graph showed the number of migrated THP1 cells, represented as relative percentage to the control (n=3).


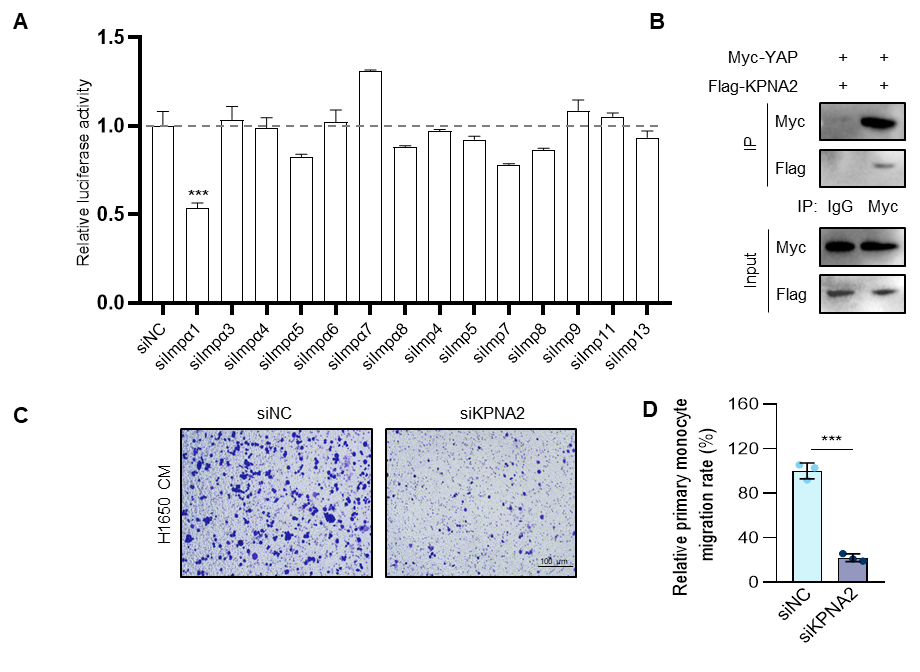


**Figure S15. Identifying the importins that affects YAP nuclear translocation.**

1. Quantification analysis of the effect of silencing the corresponding importins on YAP transcriptional activity (n=3).
2. CO-IP analysis of the interaction between YAP and KPNA2 in HEK293T cells.

C, D. Representative images and a bar graph depicted the quantity of migrated human primary monocytes stimulated by CM isolated from KPNA2 knockdown H1650 cells. The results were presented as a relative percentage compared to the control group (n = 3).


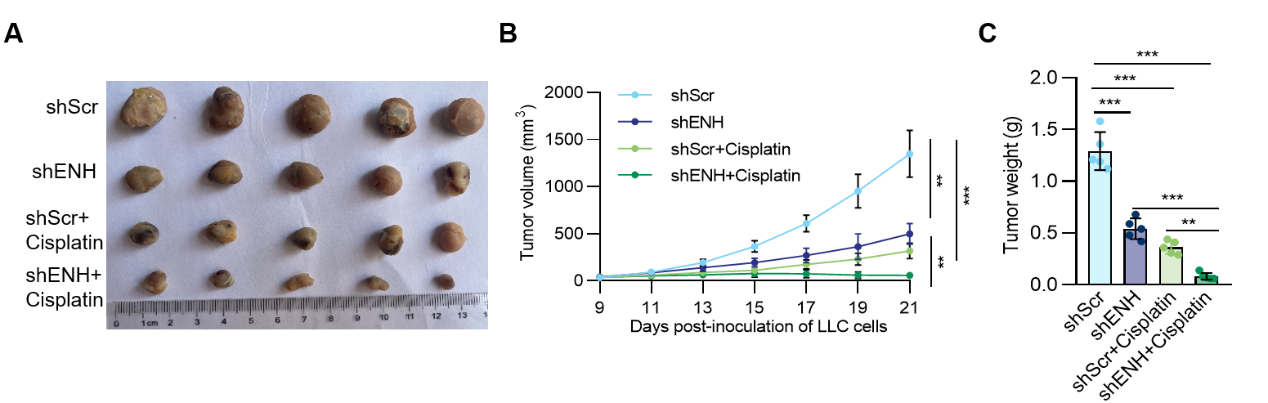


**Figure S16. ENH knockdown enhances sensitivity of LUAD to chemotherapy.**

1. Tumor tissue from each group collected 21 days after injection of LLC cells (n=5).
2. LLC-shScr and LLC-shENH tumor growth in mice treated with or without cisplatin (n=5).
3. Weight of dissected tumors obtained from mice of indicated groups (n=5).
